# Heterocyclic Anticancer Compounds: Recent Advances and the Paradigm Shift towards the Use of Nanomedicine’s Tool Box

**DOI:** 10.3390/molecules200916852

**Published:** 2015-09-16

**Authors:** Pedro Martins, João Jesus, Sofia Santos, Luis R. Raposo, Catarina Roma-Rodrigues, Pedro Viana Baptista, Alexandra R. Fernandes

**Affiliations:** 1 UCIBIO, Departamento de Ciências da Vida, Faculdade de Ciências e Tecnologia da Universidade Nova de Lisboa, Campus de Caparica, 2829-516 Caparica, Portugal; E-Mails: pf.martins@campus.fct.unl.pt (P.M.); joao.af.jesus@gmail.com (J.J.); sg.santos@campus.fct.unl.pt (S.S.); luismrraposo@gmail.com (L.R.R.); catromar@fct.unl.pt (C.R.-R.); 2 Centro de Química Estrutural, Complexo 1, Instituto Superior Técnico, Universidade de Lisboa, Av. Rovisco Pais, 1049-001 Lisboa, Portugal

**Keywords:** cancer therapy, heterocyclic compounds, oxygen and nitrogen-based heterocycles, drug delivery, nanomedicine

## Abstract

The majority of heterocycle compounds and typically common heterocycle fragments present in most pharmaceuticals currently marketed, alongside with their intrinsic versatility and unique physicochemical properties, have poised them as true cornerstones of medicinal chemistry. Apart from the already marketed drugs, there are many other being investigated for their promising activity against several malignancies. In particular, anticancer research has been capitalizing on the intrinsic versatility and dynamic core scaffold of these compounds. Nevertheless, as for any other promising anticancer drugs, heterocyclic compounds do not come without shortcomings. In this review, we provide for a concise overview of heterocyclic active compounds and families and their main applications in medicine. We shall focus on those suitable for cancer therapy while simultaneously addressing main biochemical modes of action, biological targets, structure-activity relationships as well as intrinsic limitation issues in the use of these compounds. Finally, considering the advent of nanotechnology for effective selective targeting of drugs, we shall discuss fundamental aspects and considerations on nanovectorization of such compounds that may improve pharmacokinetic/pharmacodynamic properties of heterocycles.

## 1. Introduction

With its origins rooted in organic synthesis and medicinal chemistry, heterocyclic compounds present themselves as a fundamental division of organic chemistry. Defined by IUPAC as “cyclic compounds having as ring members atoms of at least two different elements” [1], heterocycles’ ring structures are in essence composed by elements other than carbon, where the most frequent substituents are oxygen, nitrogen and sulfur [2,3]. According to the heteroatom(s) present in the ring structures, heterocycles can be classified as oxygen, nitrogen or sulfur based and, within each class, compounds are organized based on the size of the ring structure size determined by the total number of atoms [4]. The type and size of ring structures, together with the substituent groups of the core scaffold, impact strongly on the physicochemical properties [2,5]. Among the various clinical applications, heterocyclic compounds have a considerable active role as anti-bacterial [6,7], anti-viral [8], anti-fungal [9], anti-inflammatory [10], and anti-tumor drugs [11,12,13]. General applications of heterocycles are as vast as they are diverse and are not extensively encompassed in the scope of this review, hence readers are advised to refer to more detailed literature on this matter [3,14].

**Figure 1:**
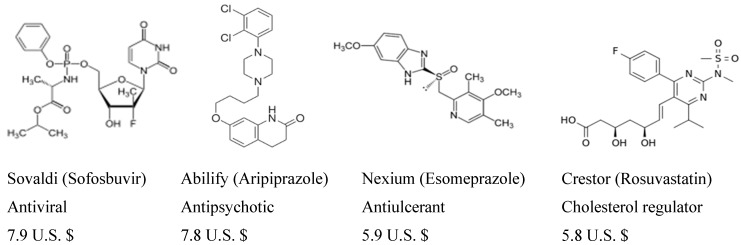
Heterocycle molecule drugs present in the US top five prescription drugs and respective retail sales in 2014 (in billions of U.S. $) [15].

Heterocycles constitute a common structural unit of most marketed drugs. Of the top five US small molecule drug retail sales in 2014, four are or contain heterocycle fragments in their overall structure (Figure 1); combined, these four account for an incredible 27.4 million U.S. dollars, almost 80% of the total revenue obtained from the top five prescription drugs [15]. 

The engineering and rationale behind drug design are closely related to the strategic incorporation of heterocyclic like fragments with specific physicochemical properties. Potency and selectivity through bioisosteric replacements, lipophilicity, polarity, and aqueous solubility can ultimately be fine-tuned to the point of altering and conditioning the possible mechanisms of action of pharmaceutical drugs in an attempt to obtain molecularly targeted agents [2]. Despite their versatility and potential, as for any other pharmaceutical, there are several issues hindering wider application and further development of such compounds into market drugs. Oncology is one of the areas where this is perhaps most noticeable, partially due to the intrinsic limitations regarding main therapeutic routes of chemotherapy, concomitant side effects and toxicity to healthy tissues. Such deleterious effects may be circumvented via selective targeting of delivery, passively or actively into cancerous cells [16]. It should be referred that for some playmakers within the chemotherapy field, the success of “molecularly targeted agents”, such as imatinib are merely fortunate exceptions and that the number of success in this area is considerably low [17]. Recent advances in interdisciplinary field of nanobiotechnology have led to the development of newly inventive therapeutic strategies and drug delivery alternatives taking advantage of the architectural geniality of systems based on nanoscale devices particularly tailored to deliver drugs to a selected tissue [18,19,20]. In this sense, nanoparticles, and the associated nanomedicine tools, are becoming the most appealing answer to chemotherapy problems, such as low drug solubility, degradation, fast clearance rates and nonspecific toxicity [21].

This review deals with the main innovations regarding nitrogen, oxygen and sulfur based heterocycle scaffolds stressing out their main roles in cancer therapy and contemplating their properties as molecule drugs, general mechanisms of action, main biological targets as well as structure–activity relationships. Innovativeness and categorization of heterocycles addressed was performed by taking into account previously approved therapies and their respective molecular drugs by assessing internal FDA databases and listed drugs from the Center for Drug Evaluation and Research (CDER) [22]. Furthermore, it tackles intrinsic problems related to the application of heterocycles in chemotherapy and provides groundwork on nanoparticle-based drug delivery systems that have proven to be therapeutically competitive with conventional drugs and therapeutic strategies.

### 1.1. Heterocycles’ Clinical Relevance in Cancer Therapy

Given the extensive structural diversity of heterocycles, a full description of all currently investigated compounds is an unfeasible task, and it should be mentioned that herein the examples depicted and addressed are based upon the most frequent ring scaffolds in FDA approved drugs [23].

#### 1.1.1. Nitrogen-Based Heterocycle

A simple glance at FDA databases reveals the structural significance of nitrogen-based heterocycles in the drug design and engineering of pharmaceuticals, with nearly 60% of unique small-molecule drugs containing a nitrogen heterocycle [24]. Recently, Edon Vitaku and colleagues comprehensively compiled the structural diversity, substitution patterns and frequency of nitrogen heterocycles among U.S. FDA approved pharmaceuticals. Noteworthy, the average number of nitrogen atoms per drug, being around 2.3, while in those containing a nitrogen heterocycle an increase to 3.1 nitrogen atoms per drug is evidenced [24]. Ultimately, the structure dynamics involved in nitrogen-based heterocycles (and other classes of heterocycles), alongside with fundamental aspects such as ring size and aromaticity, translates into a vast array of chemical structures by which their molecular mechanisms of action can vary significantly [2,5,14].

**Figure 2:**
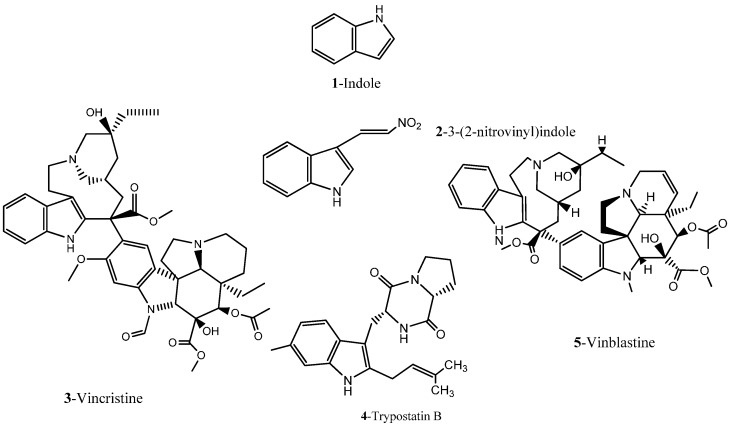
Chemical structure representation of indole basic core structure and of reported/FDA approved examples of indole like compounds as tubulin inhibitors [29].

Indoles and indole derivatives for instance comprehend one of the most versatile and common nitrogen-based heterocyclic like fragments that are frequently used in the synthesis of fundamental FDA approved drugs for common pathological conditions, ranking in the ninth position of the top 25 most frequent nitrogen heterocycles among the U.S. FDA approved drugs [24]. There has been particular emphasis on the synthesis of indole derivatives in recent decades due to the virtually endless possibilities for architectural design of polycyclic structures by the incorporation of multiple fused heterocyclic scaffolds in an attempt to achieve promising new heterocycles with chemical and biomedical relevance [25,26]. The indole structure plasticity observable in drug rational design is translated to the wide range of biological targets that these have been found to affect often ranging from topoisomerase inhibitors to G2/M abrogators and others [27]. However, application of the indole basic core structure for the synthesis of several potent tubulin polymerization inhibitors has lately been receiving increasing attention, in particular in oncology [26,27,28,29], often being present among renowned FDA approved drugs and others reported in clinical trials (Figure 2).

**Figure 3:**
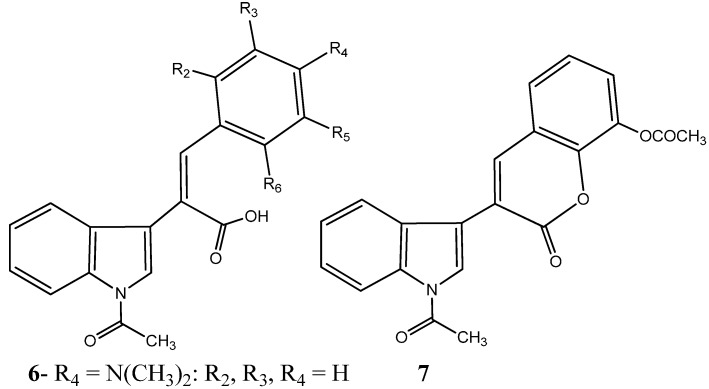
Chemical structure representation of a novel indole-bearing combretastatin analogues as tubulin polymerization inhibitors with antiproliferative activities against tumor cell lines THP1 and MCF7 [29].

In a recent study, 25 new combretastatin 2-(1-acetyl-1*H*-indole-3-yl)-3-(phenyl) propenoic analogues were evaluated for their antiproliferative properties in a small panel of tumor cell lines, including THP-1 (leukemia), A-549 (lung), IGROV-1 (ovary), HEP-2 (liver), MCF-7 (breast), and DU-145 (prostate). Among the synthetized compounds containing the indole moiety, two have been reported to elicit a significant antiproliferative activity, compound (*Z*)-2-(1-acetyl-1*H*-indol-3-yl)-3-(4-(dimethylamino) phenyl) propenoic acid (**6**) and 3-(*N*-acetyl-1*H*-indol-3-yl)-8-acetoxy-2*H*-chromen-2-one (**7**) (Figure 3) [29]. With an half maximal inhibitory concentration (IC_50_) of 0.80 and 0.37 μM in THP1 and MCF7 tumor cell lines respectively, evidenced by compound **6**, and an IC_50_ of 3.60 μM against MCF7, evidenced by compound **7**, the anti-cancer properties of these compounds were comparable to that of the conventional and clinically employed chemotherapeutic drug Paclitaxel (Taxol^®^) [29]. Moreover, the authors address the inhibition of tubulin polymerization by both compounds and their physical binding to the colchicine binding site between α and β interface of tubulin [29].

**Figure 4:**
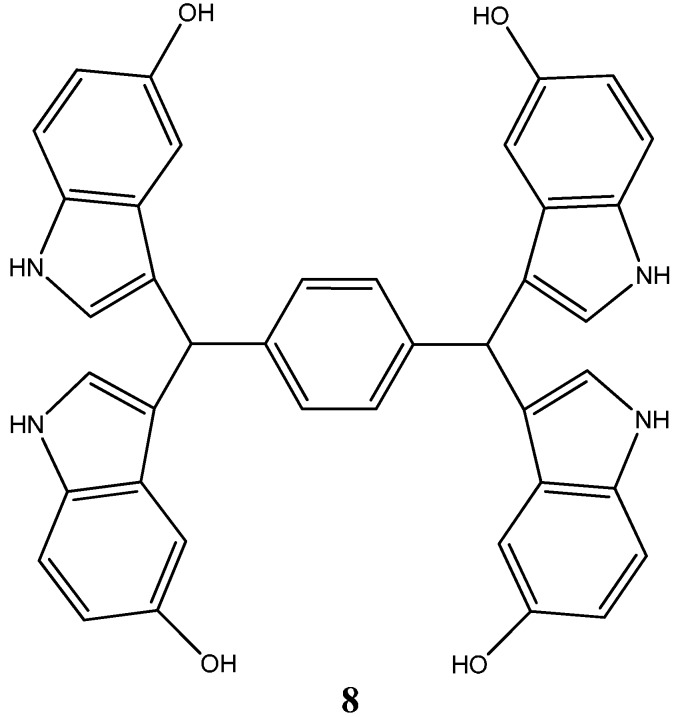
Chemical structure representation of a novel indole compound SK228 with reported antiproliferative activities through the induction of reactive oxygen species, activation of programed cell death processes and the disruption FAK/Paxillin pathway [30].

Another indole compound, 1,4-bis(di(5-hydroxy-1*H*-indol-3-yl)methyl)-benzene (SK228) (**8**) (Figure 4), was evaluated for its potential as a pharmaceutical compound for cancer treatment through a well-designed *in vitro* and *in vivo* experimentation [30]. SK228 was capable of inhibiting cell growth of human lung and esophageal cancer cell lines with proven IC_50_ values ranging from 0.28–1.86 µM. Moreover, a series of DNA interaction studies including Comet assay, DNA-intercalating assay and ROS probe showed that SK228 caused severe DNA damage in binding to the minor groove. Plus, conformational changes to plasmid DNA after exposure to increasing doses of compound, adducts and intercalation of the SK228 metabolites with nucleobases are thought to be equally responsible for its general toxicity and mutagenic potential [30]. Data relative to the induction of programed cell death mechanisms demonstrated that SK228 was able to promote phosphatidilserine flipping from the inner to the outer membrane, cytochrome *c* release from mitochondria, and ultimately caspase activation (-9; -3), which together suggest the activation of apoptosis mechanisms through the intrinsic pathway. Moreover, complementary studies (e.g., Western Blot, immunocytochemistry and trans-well invasion assays) showed that SK228 severely reduced A549 and CL15 tumor cell line’s invasion characteristics through FAK/Paxillin pathway disruption, thus providing support for SK228 clinical translation as an inhibitor of highly metastatic tumor cells [30]. Conformational changes to the linear form of the plasmid allowed to infer about the compound’s ability to intercalate within DNA through adduct formation. Adducts of such tamoxifen metabolites with glutathione and nucleobases are thought to be responsible for its general toxicity and mutagenic potential [30].

**Figure 5:**
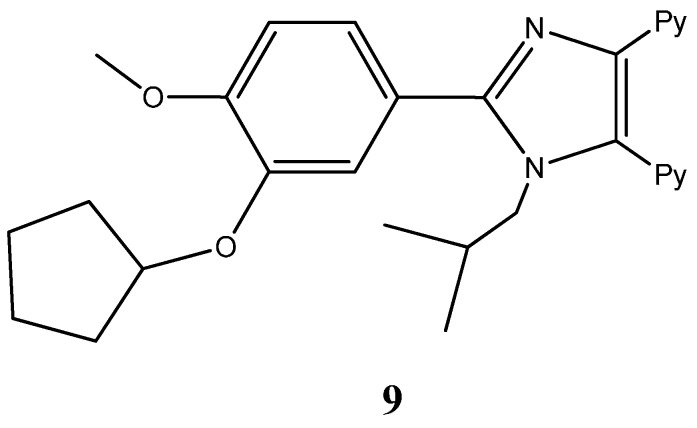
Chemical structure representation of a promising imidazole derivative with proven antiproliferative activity in A549 epithelial cancer cells, by affecting proliferation, migration, anchorage independent growth, and by inducing cycle arrest in the G2/M phase plus the activation of apoptosis [32].

Imidazole fragments have been recently attracting much attention due to their roles as attractive scaffolds for biologically active heterocyclic drugs [24,31]. In general terms, physicochemical properties like hydrogen bond donor-acceptor capability, π-π stacking interactions, co-ordination bonds with metals as a ligands, and van der Waals, polarization and hydrophobic forces have caused the increasing interest in these fragments. These properties accountable for their reactivity enable derivatives to readily bind with a series of biomolecules, including several enzymes and nucleic acids [31,32]. A vast screening study of a series of new 2,4,5-trisubstituted and 1,2,4,5-tetrasubstituted imidazoles led by Sanjay Malhotra group against the National Cancer Institute’s 60 human cancer cell line panel enabled the isolation of 2,20-(2-(3-(cyclopentyloxy)-4- methoxyphenyl)-1-isobutyl-1*H*-imidazole-4,5-diyl)dipyridine (**9**) (Figure 5) for its particular reactivity against A549 human lung cancer cell line, [32]. Further studies carried out with A549 epithelial cancer cells revealed the compound’s ability to profoundly inhibit cell proliferation, upon exposure to increasing dosages (with a proven IC_50_ of 9 μM). Most significantly, cell proliferation inhibition was found to be linked with an overall loss of cell morphology, inhibition of cell migration capabilities and the ability to induce a cell cycle arrest through senescence. Moreover, this work highlights the compound’s ability to inhibit anchorage independent growth [32].

**Figure 6:**
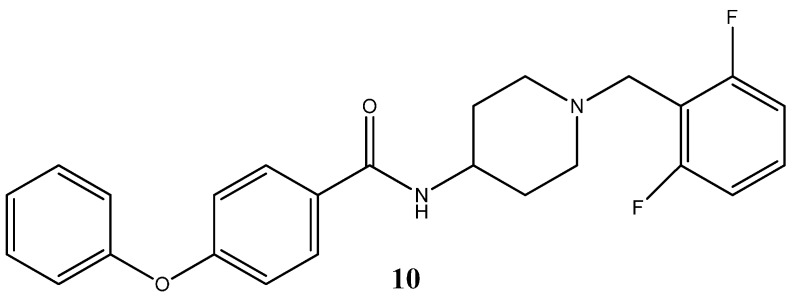
Chemical structure representation of *N*-(1-(2, 6-difluorob- benzyl)-piperidine-4-yl)-4-phenoxybenzamide complex with proven antiproliferative activity in HepG2 cells, through regulation of AMPK phosphorylation and by induction of cell cycle arrest p53/p21-dependent manner [33].

Piperidine and pyridine complexes comprehend two of the most common heterocyclic fragments present in FDA approved drugs [24]. Several *N*-(piperidine-4-yl)benzamide derivatives revealed a potent antitumor activity. For example, the structure *N*-(1-(2, 6-difluorob- benzyl)-piperidine-4-yl)-4-phenoxybenzamide (**10**) (Figure 6) was found to be the most effective against hepatocarcinoma cell line—HepG2 cells—exhibiting an IC_50_ of 0.25 μM, and the ability to regulate AMPK phosphorylation, activate downstream signaling proteins, and arrest cell cycle in a p53/p21-dependent way [33].

**Figure 7:**
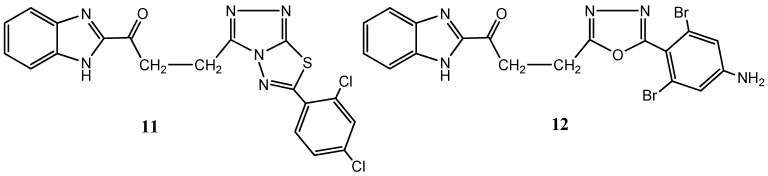
Chemical structure representation of synthetized benzimidazole hybrid heterocycles with superior selectivity for leukemia cell lines (**11**) and for non-small cell lung cancer cell lines (**12**) [35,36].

Also, among nitrogen-based heterocycles more complex scaffolds have been gaining terrain in medicinal chemistry studies over the last decade. For example, triazolothiadiazoles and triazolothiadiazines, which are cleverly designed polycyclic scaffolds arranged by combining triazoles to thiadiazoles or thiadiazines, have become important biologically relevant scaffolds in cancer [34]. Linking benzimidazole basic scaffold to other heterocyclic moieties including fused rings has led Asif Husain and colleagues to the synthesis of benzimidazole hybrid heterocycles clubbed with triazolo-thiadiazoles and triazolo-thiadiazines, in an attempt to produce improved pharmacological compounds [35]. Compound screening against several cancer cell lines has demonstrated a broad spectrum of antiproliferative activity, although among the synthetized derivatives 1-(1*H*-benzo[*d*]imidazol-2-yl)-3-(6-(2,4-dichlorophenyl)-[1,2,4]triazolo[3,4-*b*][1,3,4]thiadiazol-3-yl) propan-1-one), (**11**) (Figure 7), elicited the most significant effects with reported 50% growth inhibition (GI_50_) values ranging from 0.20–2.58 μM [35]. Previous studies from the same group have already demonstrated the same rational process by synthetizing benzimidazole heterocycles bearing oxadiazole and triazolo-thiadiazoles cores, also with proven anticancer activity. In this study, 3-(5-(4-amino-2,6-dibromophenyl)-1,3,4-oxadiazol-2-yl)-1-(1*H*-benzo[*d*]imidazol-2-yl)propan-1-one, (**12**) (Figure 7), is highlighted among the several compounds by proving to be the most active synthetized derivative, evidencing GI_50_ values ranging from 0.49–48.0 μM, against the National Cancer Institute 60 human cancer cell lines panel [36].

**Figure 8:**
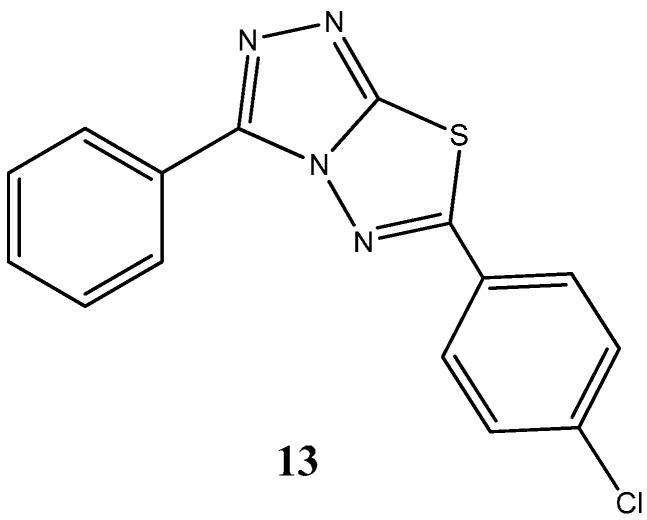
Chemical structure representation of synthetized triazolo[1,3,4]thiadiazole derivative, 6-(4-chlorophenyl)-3-(pyridin-4-yl)-[1,2,4]triazolo[3,4-*b*], with proven antiproliferative activity, with superior selectivity for gastric cancer cell lines [37].

More recently, novel 1,2,4-triazoles, triazolothiadiazines and triazolothiadiazoles have been synthetized and their anticancer potential evaluated [37]. Kamel and coworkers reported to have obtained seven compounds with considerable cytotoxic activity against a broad spectrum of cancer cell lines (NUGC; DLD1, HA22T, HEPG2, HONE1, MCF7) amongst which the compound 6-(4-chlorophenyl)-3-(pyridin-4-yl)-[1,2,4]triazolo[3,4-*b*][1,3,4]thiadiazole, (**13**) (Figure 8), presented an IC_50_ of 25 nM against gastric cancer cell line (NUGC). Noteworthy, toxicological testing employing normal fibroblast cells (WI38), in order to assess potential side effects, demonstrated significant differences, being approximately 400-fold less toxic to normal cells compared to the NUGC cell line [37].

Despite their potential, no particular correlation between the nitrogen-based heterocycle fragments and potential families of target molecules seems to exist. Nonetheless, the bulk of these fragments or pharmaceutical drugs in which they are incorporated appear to be responsible, or to take part in coordination to major biomolecules in key regulatory pathways. Tubulin coordination/inhibition, DNA cleavage, ROS induction, and cell cycle arrest through inhibition of cyclins are several possible targets that support the previous statement but are most likely not restricted to these. The perspective covered here has only taken into consideration the most recent developments regarding the application of nitrogen-based heterocycles as potential chemotherapeutic cancer drugs, given that the vast structural diversity does not allow to present all currently investigated compounds nor the majority of the nitrogen-based heterocycle scaffolds currently incorporated into renowned pharmaceuticals. For a more complete review on other major nitrogen-based scaffolds (e.g., piperidines, pyridines, piperazines, cephems, pyrolidines, pyrazoles, purines, pyrimidines, and others), their structural diversity, substitution patterns and role as fundamental components of FDA approved pharmaceuticals, the reader is referred to a more comprehensive review on this matter [24]. 

#### 1.1.2. Oxygen-Based Heterocycles

Paclitaxel (PTX, Taxol^®^), an oxygen-based heterocycle drug with an incorporated oxetane ring, has emerged as a key drug in cancer therapy [38]. The therapeutic effect of Paclitaxel is carried out by preventing the de-polymerization of microtubule polymers in a process similar to that of microtubule associated proteins (MAPs), except for the irreversible aspect of it, which leads to the progression inhibition of mitosis in cancer cells. Notwithstanding the fundamental progress that has been made in cancer therapy since the discovery of PTX, several issues still remain to be addressed. Namely, cellular alterations of tubulin structure and the amplification of efflux pumps have been strongly correlated with PTX and the acquisition of multidrug resistance profiles, burdening oncology related research and clinical treatments. Hypersensitivity, haematological and neurotoxicity are included as other equally related systemic side effects reported for PTX in literature [38]. Similarly to PTX, many other conventional chemotherapeutic drugs fit into this profile, where the therapeutic advantages are often overcome by the dreadful side effects, and hence alternatives to surpass these obstacles are required. 

**Figure 9:**
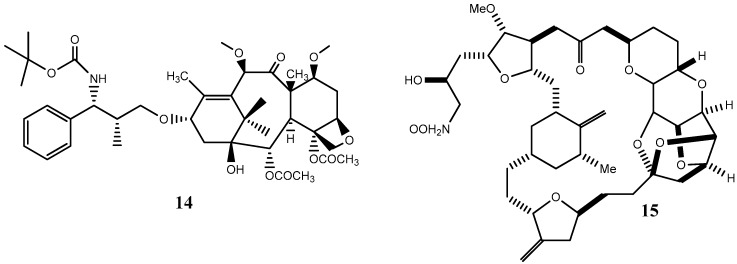
Chemical structure representation of FDA approved oxygen-based heterocycles, Cabazitaxel (**14**), with an oxetane ring, and Eribulin (**15**) with tetrahydrofuran and tetrahydropyran rings [39,40].

Since 2010, about 8% of all heterocycles with anticancer properties approved by FDA are oxygen-based heterocycles [22]. Cabazitaxel (**14**) and Eribulin (**15**) (Figure 9) were the latest drugs to be approved. 

**Figure 10:**
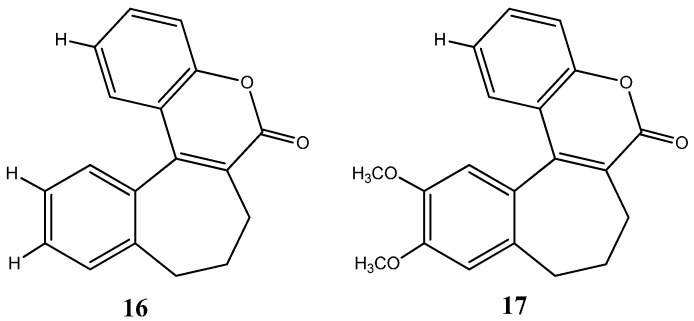
Chemical structure representation of synthetized benzosuberone derivatives bearing coumarin moieties with promising antiproliferative activity against A549, HeLa, MCF7 and MDA-MB-231 cell lines [41].

Cabazitaxel, or Jevtana^®^ its commercial name, is indicated for patient treatment with castrate resistant metastatic prostate cancer. As a taxane derivative, cabazitaxel works as microtubule inhibitor and prevents cell division [39]. Conversely, Eribulin (Halaven^®^) is a non taxane drug that acts as a microtubule inhibitor and it is used to treat patients with metastatic breast cancer [40]. Yadaguiri and coworkers synthesized different cyclic compounds bearing coumarins, oxygen-based heterocycles that showed high antiproliferative activity against several human cancer cell lines (including A549 (human alveolar adenocarcinoma cell line), HeLa (Human cervical cancer cell line), MDA-MB-231 (human breast adenocarcinoma cell line), MCF7 (human breast adenocarcinoma cell line)) compared to healthy the human cell line HEK293 (Human embryonic kidney cell line). It is particularly noteworthy that the compounds, 8,9-dihydro-7*H*-5-oxa-benzo[6,7] cyclohepta [1,2-*a*]naphthalen-6-one, (**16**) and 11,12-dimethoxy-8,9-dihydro-7*H*-5-oxa-benzo [6,7] cyclohepta [1,2-*a*]naphthalen-6-one (**17**) (Figure 10), were found to elicit promising cytotoxicity against A549, HeLa, MCF7 and MDA-MB-231. Compound **16** reported IC_50_ values ranging from 3.35–16.79 μM in the mentioned cell lines, while its counterpart, compound **17**, was found to be particularly active against HeLa and MDA-MB-231 with IC_50_ values of 6.72 and 4.87 μM, respectively [41]. As polyphenolic compounds with natural occurrence mainly in plants, these present a wide variety of modulatory and cytoprotective functions, thus offering a potential avenue for the synthesis of innovative compounds with a desirable combination of properties for anticancer drugs and superior pharmaceutical characteristics for clinical application [42,43].

Auranofin, a gold containing heterocyclic compound recognized by World Health Organization as a rheumatic arthritis therapeutic agent, was recently approved by FDA for Phase II clinical trials to treat ovarian cancer [44,45]. Auranofin which contains a tetrahydropyran ring was recently identified as a proteasomal deubiquitinase inhibitor and as a tumor growth inhibitor [45]. See-Hyoung Park and co-workers additionally suggested that auranofin activates pro-apoptotic caspase-3 in a FOXO3 dependent regulation through the induction of apoptotic proteins such as Bax and Bim and the decrease of anti-apoptotic mediator Bcl-2 in SKOV3 cell line (ovarian carcinoma cell line with a null p53 mutation) [46].

**Figure 11:**
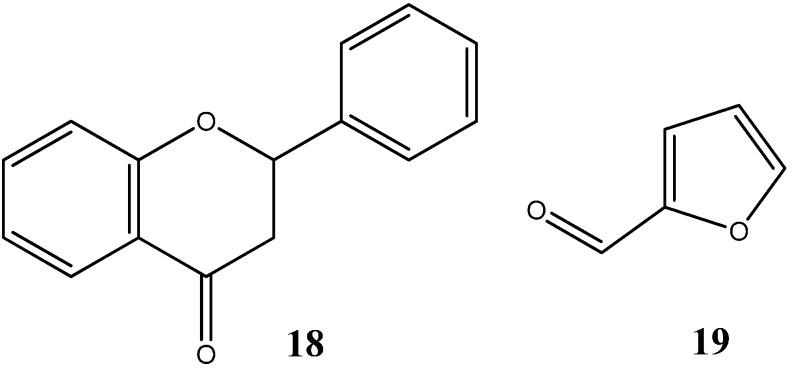
Chemical structure representation of flavanone core scaffold (**18**) and flavanone derivative, furfuraldehyde (**19**) with a reasonable cytotoxic activity against HT29, MCF7 and A498 cancer cell lines [47].

Y. Murti *et al.* showed that flavanones (**18**) (Figure 11), a natural product with extensive biological activities such as antibacterial, antifungal, analgesic and antioxidant properties with low toxicity, also possess striking anticancer activities against HT29 (human colon adenocarcinoma), MCF7 (human breast adenocarcinoma) and A498 (human kidney adenocarcinoma). Interestingly, among all the derivatives screened and analyzed, those containing furan rings (oxygen-based rings) demonstrated outstanding anticancer activity against all cell lines [47]. More notably, heterocycle flavanone derivative containing a furan ring, furfuraldehyde **19** (Figure 11), exhibited IC_50_ values of 75.9, 51.0 and 59.3μM for HT29, MCF7 and A498, respectively, thus providing pre-clinical evidence of a reasonably good anticancer compound despite the fact that the mechanism of action of this particular derivative is still uncertain. In similarity with coumarins, flavones might equally provide a sustainable building scaffold for potential new and improved pharmaceutical anticancer compounds.

**Figure 12:**
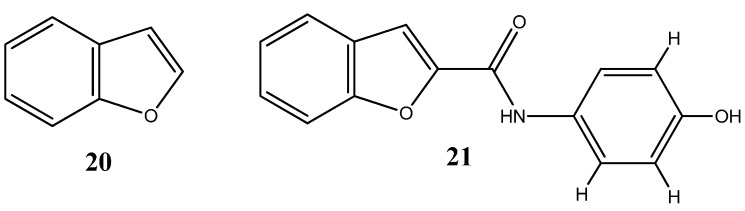
Chemical structure representation of benzofuran core scaffold (**20**) and benzofuran derivative, *N*-(40-hydroxy)phenylamide (**21**) with proven anticancer activity against HCT15, ACHN, NUGC-3, MM23, PC-3 and NCI-H23 cell lines through the inhibition of NF-κB activity [49].

Benzofurans represent another class of oxygen-based heterocycles, and as coumarins, benzofurans are equally found in nature and are known for their extensive array of biological activities [48]. Recent studies have highlighted the cytotoxic effect of benzofuran-based compounds in cancer cell lines. Minho Choi and colleagues have synthetized several benzofuran derivatives and evaluated their cell viability in several cell lines such as HCT15 (colon), ACHN (renal), NUGC-3 (gastric), MM231 (breast), PC-3 (prostate) and NCI-H23 (lung) with the purpose of developing novel scaffolds as both specific anticancer agents and inhibitors of NF-κB transcriptional activity. Results revealed high toxicities with low compound concentrations and the ability to inhibit the NF-κB transcriptional activity [49]. Perhaps most noteworthy, insights into the structure activity relationship of benzofuran derivatives, revealed that the compound *N*-(40-hydroxy)phenylamide **21** (Figure 12), was both the most cytotoxic compound among the synthetized derivatives and the most efficient at inhibiting NF-κB activity. 

**Figure 13:**
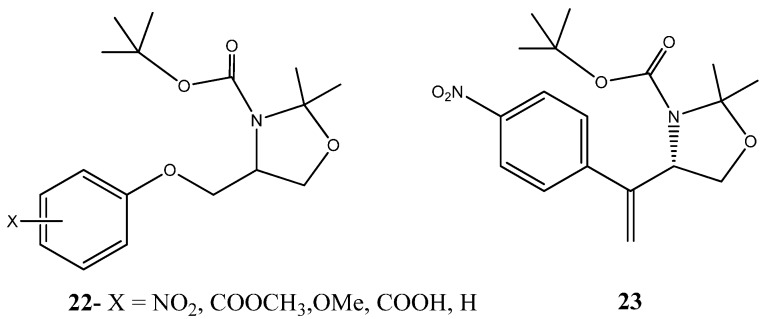
Chemical structure representation of 2,3,4-trisubstituted oxazolidines core scaffold (**22**) and oxazolidine derivative, (*S*)-tert-butyl 2,2-dimethyl-4-(1-(4-nitrophenyl)vinyl)oxazolidine-3-carboxylate (**23**) with proven anticancer activity against HL60, JURKAT, MDA-MB-231 and LNCaP [51].

Another promising heterocycles are the ones containing not only oxygen but also nitrogen in their ring structure. Felipe Rodrigues and co-workers study the antiproliferative effect of 23 different derivatives of mefloquine-oxazolidine, cyclic compounds containing oxygen and nitrogen. Following cell viability assays in HCT8 (colorectal), OVCAR8 (ovarian), HL60 (peripheral blood) and SF295 (nervous system), IC_50_ values obtained ranged from 0.59–4.79 µg/mL This was the first study to evaluate the anticancer properties of mefloquine-oxazolidine derivatives [50]. Another research performed by Saulo Andrade *et al.* analyzed oxazolidine complexes as potential anticancer agents. As a part of a previously reported work based on the core structure of a series of 2,3,4-trisubstituted oxazolidines (**22**) (Figure 13), the authors synthetized several oxazolidine derivatives with the aim of understanding the potential therapeutic effect of oxazolidine rings as building blocks for the development of chemotherapeutic cancer drugs. The rationale behind compound synthesis was based on previous observations regarding substituent groups. Interestingly, substituent groups at 3 or 4-position were found to be paramount for compound activity, and while *ortho*-substituted compounds were found to be inactive in targeting cancer cells *S* isomer were the most potent, often being 10 times more active than their enantiomers, By exploiting these characteristics and by exploiting the importance of the oxymethylene spacer between benzene and oxazolidine rings the synthetized compounds were found to exhibited in general high cytotoxic activities with low IC_50_ concentrations against a cancer cell line panel composed of, HL60, JURKAT (peripheral blood), MDA-MB-231 (mammary gland) and LNCaP (prostate) [51]. More significantly, the derivative (*S*)-tert-butyl 2,2-dimethyl-4-(1-(4-nitrophenyl)vinyl)oxazolidine-3-carboxylate (**23**) (Figure 13) exhibited a cytotoxic activity similar to the remaining derivatives, however, with exception for LNCaP cell line for which it presented high affinity with a IC_50_ value in the micromolar range (11 μM). Moreover, under the assayed conditions and given the cancer cell line panel, the activity of the compound **23** was proven to be specific for cancer cells, as it did not affect VERO or PBMC cell survival and proliferation, which in a possible translation into the clinic might suggest low side effects.

Additionally, other studies to evaluate anticancer properties of oxazolidine compounds were carried out always showing high toxicities with low IC_50_ values, thus suggesting that these families of oxygen, nitrogen-based heterocycles are a new emerging area of investigation [52,53].

The findings presented here for many of the oxygen-based heterocycles currently under pre-clinical studies indicate the occurrence of significantly different toxicities depending on the type of oxygen-based heterocycle, its overall structure, ligands, ring size and aromaticity. The evidence that oxygen-based heterocycles scaffolds have been demonstrating promising anticancer activities is most likely to be continuously exploited by pharmaceutical industries, in light of Cabazitaxel and Eribulin examples, in order to develop superior chemotherapeutic cancer drugs. Nonetheless, current research is yet to determine the exact mechanism by which many of these new heterocycle drugs/scaffolds exert their therapeutic effects.

#### 1.1.3. Sulfur-Based Heterocycles

Resulting from the derivation of homocyclic hydrocarbons from the substitution of the ring carbon atom by sulfur heteroatoms, their relevance comes from the significant changes in cyclic molecular structure engraved by differences in electron configurations, unshared pairs of electrons and ultimately the electronegativity between heteroatoms and carbon [54,55]. Physicochemical properties and reactivity of sulfur containing heterocycles are hence deeply conditioned by the overall electron configuration as well as by the versatile chemistry of the sulfur atom itself. The covalently bonded sulfur is determinant in many biological systems and is often known to form metal complexes with metal ions [56]; e.g., the proteins’ fundamental building blocks cysteine and methionine hold sulfur as key for the overall tertiary structure [57]. Other regulatory roles in biological systems range from the incorporation as a key component in several vitamin cofactors, sugars and nucleic acids to play important roles in regulating translation by sulfuration of transfer RNA [57].

**Figure 14:**
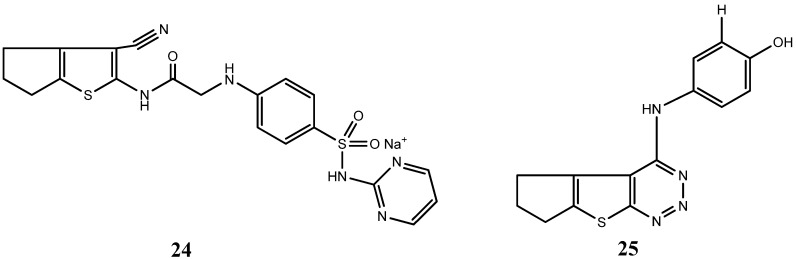
Chemical structure representation of *N*-(3- cyano-5,6-dihydro-4*H*-cyclopenta (*b*) thiophene active derivatives, *N*-(3-Cyano-5,6-dihydro-4*H*-cyclopenta[*b*]thiophen-2-yl)-2-(4-(*N*-(pyrimidin-2yl) sulfamoyl,sodiumsalt) phenylamino) acetamide (**24**) and 4-(5,6-dihydro-7*H*-cyclopenta (4:5) thieno (2,3-*d*)-1,2,3-triazin-4-ylamino)phenol (**25**) with particular antiproliferative activity for MCF7 through the inhibition of ATP recognition binding sites of tyrosine kinase receptors [58].

Given the importance of sulfur in biological systems and its increasing interest as a regulatory agent, the rationality behind the employment of sulfur-based heterocycle drugs comes to light. Said and Elshihawy synthesized *N*-(3-cyano-5,6-dihydro-4*H*-cyclopenta (*b*) thiophene derivatives and evaluated their antiproliferative activity against human breast adenocarcinoma cell line (MCF7) [58]. In this work, the newly synthetized thiophene derivative complexes, *N*-(3-Cyano-5,6-dihydro-4*H*-cyclopenta[*b*]thiophen-2- yl)-2-(4-(*N*-(pyrimidin-2yl) sulfamoyl,sodiumsalt) phenylamino) acetamide (**24**) and 4-(5,6-dihydro-7*H*-cyclopenta (4:5) thieno (2,3-*d*)-1,2,3- triazin-4-ylamino)phenol (**25**) (Figure 14), exhibited the strongest inhibitory effect with IC_50_ values of 30.8 and 38.7 nM, respectively. Furthermore, the inhibition of ATP recognition binding sites of tyrosine kinase receptors was suggested as the main possible mechanism of action by these complexes, mimicking the similar processes carried out by gefitinib and dasatinib drug molecules in the biological context [58].

**Figure 15:**
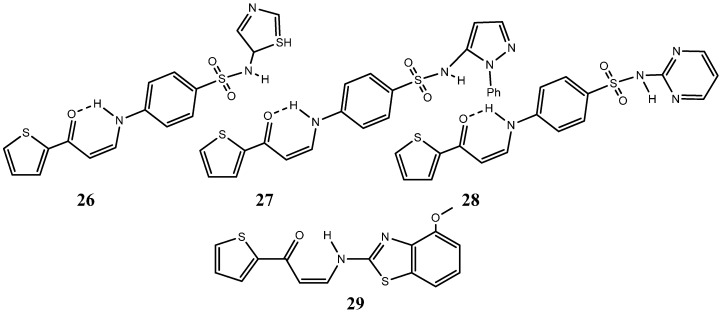
Chemical structure representation of synthetized thiophene derivatives Complexes (*Z*)-4-(3-oxo-3-(thiophen-2-yl)prop-1-enylamino)-*N*-(thiazol-2-yl)benzenesulfonamide (**26**), (*Z*)-4-(3-oxo-3-(thiophen-2-yl)prop-1-enylamino)-*N*-(1-phenyl-1*H*-pyrazol-5-yl)benzenesulfonamide (**27**), (*Z*)-4-(3-oxo-3-(thiophen-2-yl)prop-1-enylamino)-*N*-(pyrimidin-2-yl)benzenesulfonamide (**28**) and (*Z*)-3-(4-methoxybenzo[*d*]thiazol-2-ylamino)-1-(thiophen-2-yl)prop-2-en-1-one (**29**) with promising antiproliferative activity against MCF7 cell line, [59].

In a similar study, Alsaid Mansour group reported the synthesis of novel series of thiophenes having different biologically active moieties with a noteworthy antiproliferative activity comparable to that of doxorubicin [59]. Biologically active sulfonamide, isoxazole, benzothiazole, quinoline and anthracene moieties linked to the respective thiophene derivatives exhibited IC_50s_ lower than 50 µM [59]. Complexes (*Z*)-4-(3-oxo-3-(thiophen-2-yl)prop-1-enylamino)-*N*-(thiazol-2-yl)benzenesulfonamide (**26**), (*Z*)-4-(3-oxo-3-(thiophen-2-yl)prop-1-enylamino)-*N*-(1-phenyl-1*H*-pyrazol-5-yl)benzenesulfonamide (**27**), (*Z*)-4-(3-oxo-3-(thiophen-2-yl)prop-1-enylamino)-*N*-(pyrimidin-2-yl)benzenesulfonamide (**28**) and (*Z*)-3-(4-methoxybenzo[*d*]thiazol-2-ylamino)-1-(thiophen-2-yl)prop-2-en-1-one (**29**) (Figure 15), have exhibited the strongest inhibitory effect (IC_50s_ of 10.25, 9.70, 9.55, 9.39 μM respectively), approximately three times stronger than that of doxorubicin, showing great promise for a future potential drug against human breast cancer [59].

**Figure 16:**
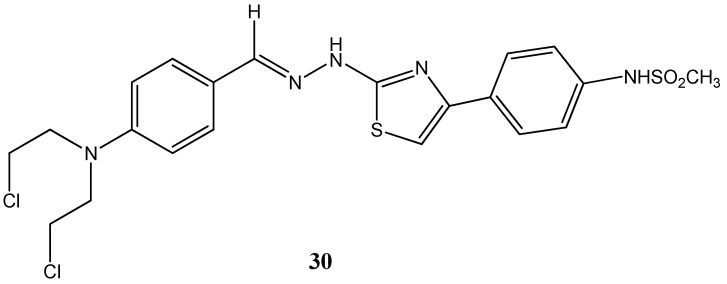
Chemical structure representation of compound (*E*)-*N*-(4-(2-(2-(4-(bis(2-chloroethyl)amino)benzylidene)hydrazinyl)thiazol-4-yl)phenyl)methanesulfonamide (**8**) with proven anticancer activity against HCT116 and MCF7 cell lines [64].

Also noteworthy, regarding sulfur-based heterocycles, are thiadiazole and thiazole complexes owing to their reactivity within the biological context. Indeed, these compounds have been found to be present in drug development for the treatment of many pathologies such as cancer, allergies, infection diseases, neurological disorders, chronic pain, fungal complications and many others [60,61,62,63]. Several new thiazole-based nitrogen mustard heterocycles have recently proven to possess strong inhibitory effects towards a panel of human cancer cell lines (MV4-11, A549, HCT116 and MCF-7). Among the several derivatives, the compound (*E*)-*N*-(4-(2-(2-(4-(bis(2-chloroethyl)amino)benzylidene)hydrazinyl)thiazol-4-yl)phenyl)methanesulfonamide, **30** (Figure 16) elicited a strong inhibitory effect in human leukemia HCT116 and MCF7 cells with reported IC_50s_ of 5.48 μM and 4.53 μM. In this study, particular emphasis was directed towards the evaluation of the cytotoxicity of these derivatives in healthy cells. Furthermore, quantum chemical calculations and binding interaction studies showed that the stronger interaction with DNA is observed for compound **30** compared with the other compounds. Moreover, *in silico* analysis demonstrated the potential of the compounds to exert their action through the inhibition of human topoisomerase II [64].

**Figure 17:**
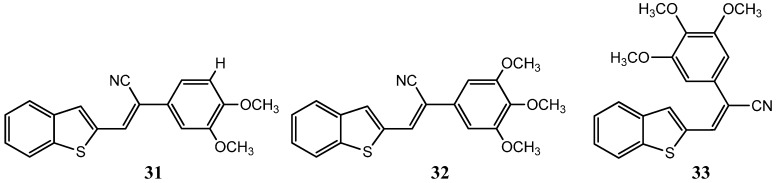
Chemical structure representation of synthetized benzothiophene acrylonitrile derivatives, ([*Z*-3-(benzo[*b*]thiophen-2-yl)-2-(3,4-dimethoxyphenyl)acrylonitrile] (**31**), [*Z*-3-(benzo[*b*]thiophen-2-yl)-2-(3,4,5-trimethoxyphenyl)acrylonitrile] (**32**) and [*E*-3-(benzo[*b*]thiophen-2-yl)-2-(3,4,5-trimethoxyphenyl)acrylonitrile]) (**33**) with promising antiproliferative activity [65].

Benzothiophene acrylonitrile derivatives, similar in structure to the natural combretastatins, have equally proven to be interesting scaffolds towards the synthesis of novel anticancer compounds with improved pharmacological profiles, as Peter Crooks has elegantly demonstrated [65]. Analog screening against 60 human cancer cell lines revealed three potent anticancer compounds ([*Z*-3-(benzo[*b*]thiophen-2-yl)-2-(3,4-dimethoxyphenyl)acrylonitrile] (**31**), [*Z*-3-(benzo[*b*]thiophen-2-yl)-2-(3,4,5-trimethoxyphenyl)acrylonitrile] (**32**) and [*E*-3-(benzo[*b*]thiophen-2-yl)-2-(3,4,5-trimethoxyphenyl)acrylonitrile]) (**33**) (Figure 17) with GI_50_ ranging from <10 to >100 nM [65]. In addition, long term acquired resistance commonly associated with many forms of tumor progression due to the overexpression P-glycoprotein (P-gp) efflux pumps seems to have been surpassed by the use of these analogues [65].

### 1.2. Approved Molecular Entities

The presence of heterocycles in a wide range of compounds with biologically and pharmacologically interesting properties is a testimony to why its relevance is undeniable in the field of medicinal chemistry. By the end of the second millennium, out of all of the 20 million chemical compounds documented in the literature, approximately half were heterocyclic [54]. Indeed, each year new molecular structures are documented, as well as new medication drugs that contribute to the quality of care and relieve the world’s burden of most common diseases. In an effort to provide a concise overview on the world’s most recent medication approvals (2010–2015) and their clinical relevance in cancer therapy, we assessed the internal FDA and CDER databases retrieving fundamental oncology related data. Between 2010 and 2015, 26 entries with heterocyclic drug names approved by FDA for their anti-tumor properties discriminated by class, drug name, bioactive compound, therapeutic indication and approval dates provide a glimpse of novel drug formulations emphasizing those that offer innovative treatments to patients—see Table 1 [22]. For instance, Zydelig^®^ (idelalisib) approved in 2014, offered, chronic lymphocytic leukemia patients a new avenue for treatment by inhibiting phosphoinositide 3-kinase delta (PI3Kδ), consequently leading to lymph node shrinkage and increased rates of apoptosis (lymphocyte destruction) [66,67]. Further studies have associated Zydelig^®^ therapeutic action to the disruption of integrin-mediated cell adhesion by the phosphorylation inhibition of Akt [67].

Another pharmaceutical drug worth mentioning is Ibrance^®^ (Palbociclib), for its particular mechanism action towards breast cancer, the world’s second most deadly form of cancer [68,69]. Palbociclib is significantly active in breast cancer models by selectively inhibiting cyclin-dependent kinases 4 and 6, important regulators in the intricate molecular network of cell cycle progression [69], blocking sustained tumor growth and suppressing DNA replication. Moreover, Phase I studies have shown that palbociclib is well tolerated.

Other novel approved heterocycle anticancer drugs are depicted in Table 1. Further information regarding their main mechanisms of action are reported elsewhere [22].

**Table 1:** Heterocyclic drug names approved by FDA for their anti-tumor properties (2010–2015). Heterocycles are discriminated by class, drug name, bioactive compound, therapeutic indication and approval dates.

Drug Name (Company)	Chemical Structure	Bioactive Compound	Therapeutic Indication	Approval Date
Approved Nitrogen-Based Heterocycle Drugs				
Xalkori^®^ (Pfizer)	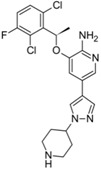	Crizotinib	Late-stage Non-small-cell lung carcinoma (NSCLC)	2011
Zelboraf^®^ (Hoffmann-La Roche)	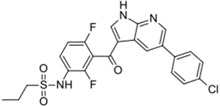	Vemurafenib	Metastatic or unresectable melanoma	2011
Zytiga^®^ (Centocor Ortho Biotech)	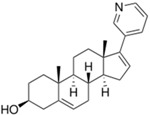	Abiraterone acetate	Metastatic castration-resistant prostate cancer	2011
Caprelsa^®^ (AstraZeneca)	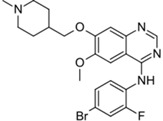	Vandetanib	Metastatic medullary thyroid cancer	2011
Iclusig^®^ (ARIAD Pharmaceuticals)	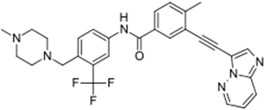	Ponatinib	Chronic myeloid leukemia/lymphoblastic leukemia	2012
Cometriq^®^ (Exelixis)	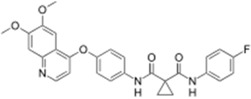	Cabozantinib	Metastasized medullary thyroid cancer	2012
Stivarga^®^ (Bayer HealthCare)	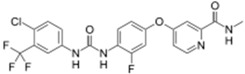	Regorafenib	Metastatic colorectal cancer	2012
Bosulif^®^ (Pfizer)	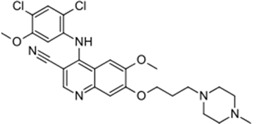	Bosutinib	Chronic myelogenous leukemia	2012
Xtandi^®^ (Astellas Pharma)	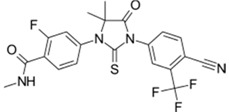	Enzalutamide	Metastatic castration-resistant prostate cancer	2012
Erivedge^®^ (Genentech)	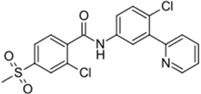	Vismodegib	Basal cell carcinoma	2012
Inlyta^®^ (Pfizer)	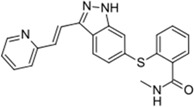	Axitinib	Renal cell carcinoma	2012
Imbruvica^®^ (Pharmacyclics/Janssen Biotec)	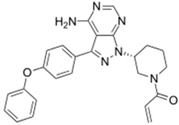	Ibrutinib	Mantle cell lymphoma	2013
Pomalyst^®^ (Celgene)	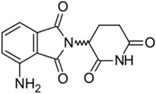	Pomalidomide	Multiple myeloma	2013
Lynparza^®^ (AstraZeneca)	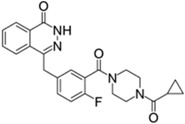	Olaparib	Advanced ovarian cancer	2014
Zydelig^®^ (Pharmacyclics/Janssen Biotec)	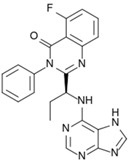	Idelalisib	Chronic lymphocytic leukemia	2014
Zycadia^®^ (Novartis)	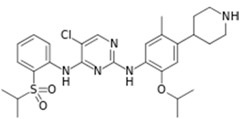	Ceritinib	Metastatic NSCLC	2014
Farydak^®^ (Novartis)	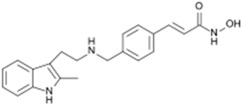	Panobinostat	Multiple myeloma	2015
Lenvima^®^ (Eisai)	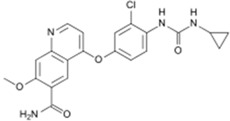	Lenvatinib	Progressive and differentiated thyroid cancer	2015
Ibrance^®^ (Pfizer)	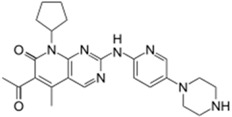	Palbociclib	Metastatic breast cancer	2015
Approved Oxygen-Based Heterocycle Drugs				
Jevtana^®^ (Sanofi-aventis)	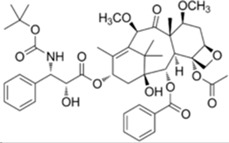	Cabazitaxel	Metastatic prostate cancer	2010
Halaven^®^ (Eisai)	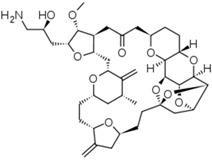	Eribulin mesylate	Metastatic breast cancer	2010
Approved Nitrogen, Oxygen-Based Heterocycle Drugs				
Synribo^®^ (Frazer)	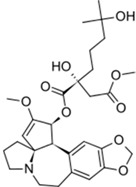	Omacetaxine mepesuccinate	Chronic myelogenous leukemia	2012
Kyprolis^®^ (Onyx)	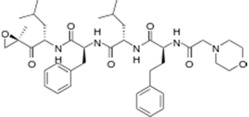	Carfilzomib	Multiple myeloma	2012
Gilotrif^®^ (Boehringer Ingelheim)	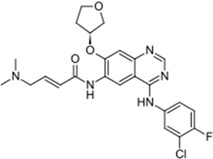	Afatinib	Metastatic NSCLC (EGFR mutations)	2013
Mekinist^®^ (GlaxoSmithKline)	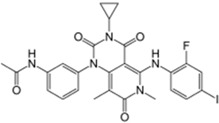	Trametinib	Tumors that express the BRAF V600E or V600K gene mutations	2013
Approved Nitrogen, Sulfur-Based Heterocycle Drugs				
Tafinlar^®^ (GlaxoSmithKline)	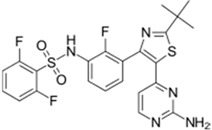	Dabrafenib	Melanoma that express the BRAF V600E gene mutation	2013

Note: Information obtained through FDA and CDER internal dataset compilation; Chemical structures were obtain from PubChem Compound http://www.fda.gov/Drugs/DevelopmentApprovalProcess/DrugInnovation/default.htm [22]; http://www.ncbi.nlm.nih.gov/pccompound [70].

**Figure 18:**
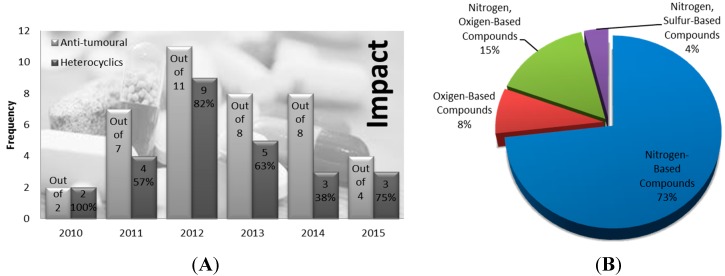
Impact of heterocyclic FDA approved anti-tumor drugs during the calendar years of 2010–2015 (**A**) and their respective discrimination by class and frequency (**B**) [22].

Ultimately, the annual number of approved compounds is greatly influenced and determined by the number of drug applications submitted. Over the past five years, the number of submission applications for new molecular drug entities by pharmaceutical industry has varied greatly with no specific trend observed. Of the 40 novel molecular anticancer entities approved during this time period, 26 are or contain heterocycle fragments within their molecular composition, representing a total of 65%. Also noteworthy during the year 2012, out of 11 anticancer drugs, nine—or approximately 82%—were found to be heterocycles in nature, ranking it as one of the most productive years regarding heterocycle medicinal chemistry. On the other extreme, only 38% of the total 11 anti-tumoral compounds in 2014 were found to be heterocycles (Figure 18A). Furthermore, among the heterocyclic anti-tumor drugs, we decided to measure the frequency of each heterocycle class in order to determine the relevance of their incorporation in current pharmaceuticals as scaffolds for biologically active fragments (Figure 18B). The current blockbuster regarding the incorporation of heterocycles in current chemotherapeutic drugs is clearly dominated by nitrogen-based heterocycles comprehending a total of 73%. This incredibly high percentage far surpasses the numbers evidenced for nitrogen-oxygen based heterocycles (15%), the second most common class. Oxygen-based and nitrogen-sulfur-based heterocycles follow, representing 8% and 4% of the total, respectively. The rationality behind the main use of oxygen, sulfur and especially nitrogen, stems from the fact that most drug pipeline research is deeply based on mimicking nature’s compounds and key regulators [2].

This brief overview offered a perspective on the most recent and most common heterocycles fragments present in anticancer pharmaceuticals since 2010, but one should keep in mind that the most important remains the efficacy of novel drugs and their impact on public health. The ongoing application of heterocycles is a clear sign of their importance for the pharmaceutical industry due to their unique contributions to medical care and public health. 

However, inherent to the synthesis of new compounds, either heterocyclic or not, their rational design and overall engineering conceptualization is always subject to many considerations including their pharmacodynamic and pharmacokinetic profiles as well as their toxic side effects. For an effective clinical translation, these must all be assessed and duly analyzed.

### 1.3. Drug Design and Structure–Activity Relationship

Drug pipeline bottlenecks are often associated with pharmacokinetic hurdles deeply related to the significant lack of structural and functional information available for new drug targets. In other words, the promising therapeutic action of many potential new drugs is often compromised due to limited information regarding their physicochemical characteristics, solubility and stability hindrances and biodistribution parameters. As a result, the unpredictability of preclinical drugs is often the norm rather than the exception [2,5]. The rational design of heterocycle compounds and activity relationships studies tackle problems inherent to this issue such as absorption, distribution, metabolism, excretion and toxicity parameters of the drug target in question as well as poor solubility and cellular uptake mechanisms [5]. Moreover recently, with the advent of *in silico* approaches and the significant improvement of crystallography and NMR databases, the available information regarding compound stability and other relevant chemical information as well as the prediction of a wide number of interesting molecular targets for structure-based drug design is available to us at the touch of a button [71,72].

The basis for heterocycle engineering and rational design is intricately connected to the wide range of heterocyclic structures present in the biological systems, ranging from co-factors to nucleic acids and proteins. Typical drug modeling employing heterocyclic structures tends to follow “nature’s guidelines” in an attempt to mimic nature’s heterocycle compounds and exert their therapeutic effect by deceiving nature’s regulatory pathways [2]. Furthermore, from a rational design point of view, reactivity, targeting and molecular docking, lipophilicity, polarity, and solubility encompass the fundamental drug like properties that ultimately can be fine-tuned by the inclusion of heterocyclic moieties in order to improve the pharmacodynamic and pharmacokinetic profiles of a specific drug [2].

**Figure 19:**
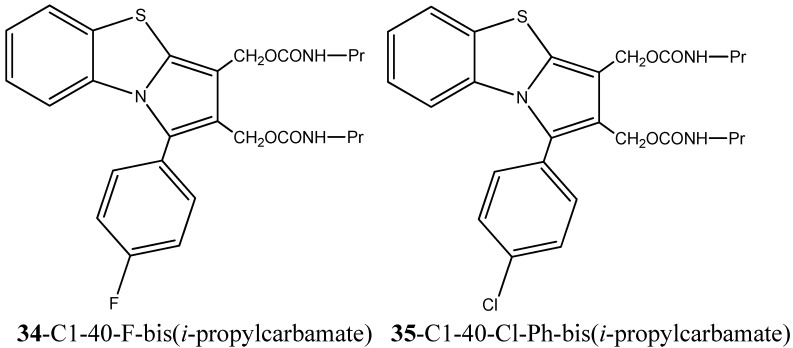
Benzo[*d*]pyrrolo[2,1-*b*]thiazole with bis(*i*-propylcarbamates) derivatives with proven cytotoxic activity against lymphoblastic leukemia CCRF-CEM and human breast carcinoma MX-1 xenografts [73].

Reports on a series of novel 2,3-bis(hydroxymethyl)benzo[*d*]pyrrolo[2,1-*b*]thiazoles and their bis(alkylcarbamate) derivatives, described by Ravi Chaniyara *et al.*, highlight the importance of substituent heterocyclic fragments in drug modulation and in the enhancement of pharmacological properties. In particular, structure–activity relationship studies have shown that the bis(alkylcarbamates) derivatives influence greatly the cytotoxicity against human lymphoblastic leukemia CCRF-CEM and other tumor cell lines when compared with its counterpart bis(hydroxymethyl). In fact, the application of C1-40-F- or C1-40-Cl-Ph-bis(*i*-propylcarbamates) derivatives, **34**- [1-(4-Fluorophenyl)benzo[*d*]pyrrolo[2,1-*b*]thiazole-2,3-diyl] bis(methylene)bis(iso-propylcarbamate) and **35**- [1-(4-Chlorophenyl)benzo[*d*]pyrrolo[2,1-*b*]thiazole-2,3-diyl]bis(methylene)bis(iso-propylcarbamate) respectively (Figure 19), in *in vivo* studies to assess the remission potential of human breast carcinoma MX-1 xenografts has proven to be significantly effective, inducing complete tumor remission. Moreover, the authors report DNA interstrand cross-linking as the main bis(alkylcarbamates) derivatives’ mechanism of action [73].

Another study includes a set of triazoles with fused-heterocycle fragments that were designed and evaluated for their reactivity against fungal [9]. Drug modulation was performed on the basis of structure–activity relationship studies and on the binding mode of albaconazole. Data analysis on tetrahydro-[1,2,4]-triazolo[1,5-*a*]pyrazine and tetrahydro-thiazolo[5,4-*c*]pyridine revealed these attained improved pharmacological profiles. The incorporation of nitrogen-based aromatic heterocycles was shown to elicit increased potency, a broader spectrum of activity and it more significantly increased rates of hydrosolubility when compared with other fused-heterocycles used in this study [9]. Together, these studies provide evidence on how the use of heterocyclic substituent groups can condition favorable pharmacokinetic profiles.

Despite the central role played by heterocycles in drug design and rational drug engineering, new molecular drugs must prove not only their ability to reach the target site of action but equally prove their therapeutic effect. Rational design offers a possible avenue for tinkering with psychochemical characteristics and, in the end, with pharmacological properties of drugs, despite being only to a certain degree. Most often, poor drug physicochemical properties overcome the desired therapeutic effects, hindering and clogging the drug pipeline discovery process. Notwithstanding the fundamental progress that has been made in cancer therapy through the use of heterocyclic chemistry, many of the current clinically applied chemotherapeutic drugs still face many hardships regarding their pharmacokinetic/pharmacodynamic properties and off-target effects, opening new opportunity avenues for improvement through drug design/(nano)vectorization strategies. The increased interest in nanoparticle drug delivery systems over the last decades emerges in this context as an answer to common setbacks regarding heterocyclic compound formulation issues [18].

## 2. Nanomedicine for Heterocyclic Compound Vectorization in Cancer 

As means to advance the use of heterocyclic compounds toward clinical translation, several formulation studies have been performed using several nanocarrier systems with the mind set of bypassing heterocycles’ poor drug physicochemical properties, including pharmacokinetic and pharmacodynamic characteristics.

At present, there are 30 main drug delivery products (Table 2) on the market with a total annual income of US$33 billion and an annual growth of 15% (based on global product revenue). The global market trend for nanoparticles (NPs) in biotechnology, drug development and drug delivery was valued at US$17.5 billion in 2011 and is expected to reach US$53.3 billion in 2017 [21,74].

The ability to bind and/or encapsulate already tested and approved heterocyclic compounds into nanocarriers allows the exploit of the enhanced permeability and retention effect suitable for tumor targeting, or passive targeting. This allows for an increase of concentration at the tumor site with a consequent increase of efficacy while decreasing side effects and the destruction of healthy tissues. Additional targeting moieties may be conjugated at the surface of the nanocarriers—active targeting—improving selectivity towards cancer cells with specific overexpressed receptors, and/or other biomarkers [75]. Nanovectorization systems include liposomes, polymeric nanoparticles, albumin bound nanoparticles, metallic nanoparticles and Dendrimers [76]. Despite the wide variety of available nanodevices, there is a tendency favoring encapsulation, mainly in liposomal formulations, with 29 nanoformulations in ongoing clinical trials [77].

### 2.1. Nanoparticle Diversity

#### 2.1.1. Liposomes

Liposome is a phospholid system comprising a bilayer structure, and since its early studies has been described as a potential drug delivery system [78]. Since its discovery, the synthesis method has seen much improvement, allowing a more large scale production and smaller sizes, from 20–50 nm. Liposomal doxorubicin (Doxil^®^) was the first nanodevice approved by FDA for cancer treatment [79,80], and an additional five formulations have already attained approval—DaunoXome^®^, Myocet^®^, Marqibo^®^, Lipo-Dox^®^ and DepoCyt^®^ [80,81].

In terms of drug loading, liposomes not only encapsulate water soluble drugs but can also encapsulate hydrophobic compounds inside their membranes [82]. However, the latter, due to the small amount of room available between their bilayer structure, and the destabilization effect in the outer membrane, is limited [81]. It is possible to increase drug loading in response to pH gradients that favors weak bases drugs encapsulation, which represents the majority of cancer drugs. For the remaining drugs, that do not precipitate inside liposome and are difficult to retain, one surpasses the limit of solubility to force precipitation, or turning such drugs, for example Paclitaxel, into a weak base drug [80]. Liposomes are mainly considered for water soluble drugs [81]. Most liposome formulations are biocompatible and almost biologically inert, with great circulation time, but intravenous injection seems to be associated to complement activation-related pseudo-allergy [81,83]. To increase the circulation time in blood, liposomes may be conjugated with PEG, allowing to bypass the immune system, primarily evading opsonization. Liposomes may be conjugated with targeting moieties, e.g., antibodies, forming immunoliposomes. Despite the potential advantages of active targeting, thus far, no immunoliposome has attained FDA approval, and only a few are in clinical trials—MM-302, a ErbB2/ErbB3-targeted liposomal doxorubicin, and, MBP-436, a transferrin-targeted liposomal oxaliplatin [80].

#### 2.1.2. Polymeric Nanocarriers

Polymeric nanocarriers are polymer-based structures, such as polymeric micelles, dendrimers and polymeric nanoparticles, acting as an alternative to liposomes, but with more *in vivo* stability, higher drug circulation time and loading, and a more controlled release profile [81].

Polymeric micelles are composed by amphilic block copolymers, forming a sphere-like structure with micelles and a hydrophobic core [84]. The core can contain drugs with poor water solubility and the micelles allow inclusion of hydrophilic drugs, which provides stability to the micelles themselves [85]. Directed targeting of tumors and stimuli-responsive approaches, drug entrapment into the core and functionalization with targeting moieties are also being developed. Currently, there are only eight polymeric micelle formulations in ongoing clinical trials [81].

Dendrimers are hyperbranched polymeric molecules ranging from 10–100 nm [85,86]. Dendrimer syntheses are performed from a central core followed by consecutive controlled polymeric reactions yielding a highly controlled architecture and tuning of pharmacokinetic parameters [85,86,87]. Anticancer drugs may be non-covalent encapsulated into the core or covalent conjugated to the dendrimer surface, modulating the drug release profile [85,86]. Non-covalent encapsulation in the core is reported to cause toxicity and an uncontrolled release profile, and is only used in intratumor administration [81].

**Table 2:** Nanoscale drug delivery devices, FDA approved or in different phases of clinical trials by which the bioactive compounds mentioned herein are heterocyclic in nature.

Name	Formulation	Target Ligand	Bioactive Compound	Indication	Status	Ref.
DaunoXome^®^	Non-PEGylated liposomes	None	Daunorubicin	Kaposi’s sarcoma	Approved	[77,80,88]
Myocet^®^	Non-PEGylated liposomes	None	Doxorubicin	Breast cancer	Approved	[77,80,88]
Depocyt^®^	Non-PEGylated liposomes	None	Cytarabine	Leukemia; Glioblastoma	Approved	[77,80,88]
Doxil^®^/Caelyx^®^	PEGylated liposomes	None	Doxorubicin	Breast cancer; ovarian cancer; multiple myeloma; Kaposi’s sarcoma	Approved	[77,80,88]
Thermodox^®^	PEGylated liposomes	None	Doxorubicin	Liver cancer; breast cancer	Phase III	[88]
NK105	PEG-poly(aspartic acid)	None	Paclitaxel	Breast cancer	Phase III	[88]
Opaxio™	PGA-paclitaxel	None	Paclitaxel	Lung cancer; ovarian cancer	Phase III	[77,88]
NC-6004	PEG-poly(glutamic acid)	None	Cisplatin	Pancreatic cancer	Phase II/III	[77,88]
Abraxane^®^	Albumin-based	None	Paclitaxel	Breast cancer	Approved	[77,88]
Paclical^®^	Micellar retinoid-derived	None	Paclitaxel	Ovarian cancer	Phase III	[77,88]
Oncaspar^®^	PEG-l-asparaginase	None	Asparagine specific enzyme	Acute lymphoblastic leukemia	Approved	[77,88]
Lipo-Dox	PEGylated liposomes	None	Doxorubicin	Kaposi’s sarcoma; breast cancer and ovarian cancer	Approved	[77,80]
Marqibo	Non-PEGylated liposomes	None	Vincristine	Acute lymphoblastic leukemia	Approved	[77,80]
CPX-351	Non-PEGylated liposomes	None	Cytarabine:daunorubicin	Acute myeloid leukemia	Phase II/III	[77,80]
MM-398	Non-PEGylated liposomes	None	CPT-11	Gastric and pancreatic cancer	Phase III	[77,80]
Lipoplatin	Non-PEGylated liposomes	None	Cisplatin	Non-small cell lung cancer	Phase III	[77,80]
ThermoDox	Non-PEGylated liposomes	None	Thermosensitive doxorubicin	Primary hepatocellular carcinoma	Phase III	[77,80]
Stimuvax	Non-PEGylated liposomes	None	Anti-MUC1 cancer vaccine	Non-small cell lung cancer	Phase III	[77,80]
Mylotarg^®^	Antibody drug conjugate (Gemtuzumab ozogamicin)	CD33	Calicheamicin	Acute myeloid leukemia	Approved	[77]
Adcetris^®^	Antibody drug conjugate (Brentuximab vedotin)	CD30	MMAE	Non-Hodgkin lymphoma	Approved	[77]
Kadcyla^®^	Antibody drug conjugate (Trastuzumab emtansine)	HER2	DM1	Breast cancer	Approved	[77]
CMC-544	Antibody drug conjugate (Inotuzumab ozogamicin)	CD22	Calicheamicin	Acute lymphoblastic leukemia	Phase III	[89]
CMA-676	Antibody drug conjugate (Gemtuzumab ozogamicin)	CD33	Calicheamicin	Aute Myeloid Leukemia	Phase III	[89]
Genexol-PM^®^ (IG-001)	PEGylated liposomes	None	Paclitaxel	Breast Cancer; Lung Cancer	Approved	[77]
Mepact^®^	Non-PEGylated liposomes	None	Mifamurtide	Osteosarcoma	Approved	[77]
Zinostatin stimalamer^®^	Polymer protein conjugate	None	Styrene maleic anhydride neocarzinostatin (SMANCS)	Liver cancer, renal cancer	Approved	[77]
NKTR-102 (Etirinotecan pegol)	PEG drug conjugate	None	Irinotecan	Breast cancer; Ovarian Cancer; Colorectal Cancer	Phase III	[77]
Taxoprexin	Docosahexaenoic acid drug conjugate	None	Paclitaxel	Melanoma; Liver cancer; Adenocarcinoma; Kidney Cancer; Non-small-cell lung cancer	Phase II/III	[77]
Lipusu	Non-PEGylated liposomes	None	Paclitaxel	Solid tumors; Metastatic Breast Cancer	Phase IV	[77]

#### 2.1.3. Albumin Bound Nanoparticles

Due to the amino and carboxyl groups present in albumin, it is capable of forming covalent bonds with drugs and encapsulating them [77,78,90]. In addition, albumin is biodegradable, biocompatible, and considered non-toxic and non-immunogenic, which is optimal for synthesis of nanoparticles [78]. Albumin bound nanoparticles’ (nab) technology is an ideal platform to encapsulate lipophilic drugs. Despite the advantages, only one nab has been approved by FDA and is currently in clinical practice—nab-paclitaxel (Abraxane^®^) [78]. 

#### 2.1.4. Metallic Nanoparticles

Among all nanoparticles systems used in cancer research, these may be the more versatile model, showing applicability in, from enhanced radiotherapy, tumor imaging and drug delivery [77]. The most widely used metallic nanoparticles are superparamagnetic ion oxide nanoparticles (SPIONs) and gold nanoparticles. 

SPIONs are mainly used for imaging (magnetic resonance imaging, MRI) and diagnostic purposes. The only metallic nanoparticle approved for cancer therapy, NanoTherm^®^, is a good example of a SPION [77]. 

Gold nanoparticles (AuNPs) have become an efficient platform for diagnosis and imaging (CT scanning), selective drug delivery and for direct treatment by hyperthermia. One of the greatest features of AuNPs is the diversity available in shape and size that may be easily tuned for conjugation to biomolecules of interest. Their capability to generate heat upon near-infrared excitation is a promising approach for directed hyperthermia [91]. To date, no metallic nanoparticle for drug delivery has been approved. Some studies show an efficient binding capacity of drugs, for example paclitaxel has been known to form conjugates with gold nanorods acting as a drug nanocarrier and as a photothermal agent [92]. 

#### 2.1.5. Drug Conjugates

Drug conjugates, nanosized carriers in cancer therapy and diagnosis, comprehend both polymer-based and antibody-drug conjugates, however, here we focus mainly on the latter. The antibody-drug conjugate (ADC) concept dates back to the 1980s [89,93], where a drug was combined directly to a targeting agent such as an antibody. A second-generation ADC emerged with improved linker technology and better targeting knowledge [94]. The primary feature of these nanostructures is the reduced side effects as an intrinsic characteristic. The mechanism of action comprises the targeting of cancer cells with a certain biomarker by a corresponding antibody, and, consequently, internalization of the nanoconjugate. An expanded therapeutic window from its specific targeting explains the reduced side effects of these nano-systems [95].

Several antibody-drug conjugates have been and are continuing to be approved by FDA. The last entities recognized by this organization as drugs with anticancer properties were Dinutuximab (Unituxin^®^) in 2015 and Nivolumab (Opdivo^®^) in 2014. Dinutuximab is indicated for pediatric high-risk neuroblastoma and targets GD2, a glycolipid overexpressed on the surface of neuroblastoma cancer cells [96]. Nivolumab was approved to treat patients with unresectable or metastatic melanoma [97]. Studies addressed by Caroline Robert and coworkers reported enhanced survival ratios in overall patients with metastatic melanoma without BRAF mutation [98].

Antibody-drug conjugates are a thriving area of investigation and surely are attracting pharmaceutical companies with flourishing ideas in order to improve linker technologies or antibody stability. In combination with clinical results, ADC technology is a growing field with a promising future [93]. The scope of this review does not focus on this particular nano-scale concept, and so, for further insights readers are advised to view the following literature [95,99,100,101].

### 2.2. From Bench to Bedside

**Figure 20:**
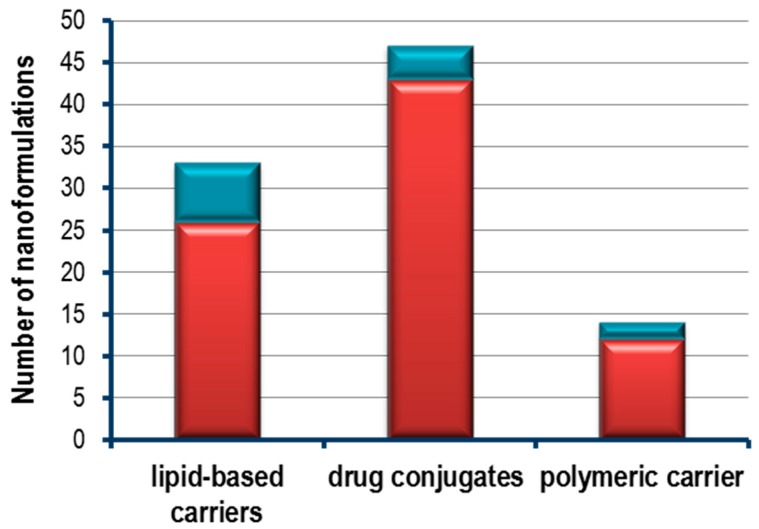
Number of heterocyclic compounds nanoformulations currently in clinical trial (red bars) and FDA approved (blue bars) for cancer therapy. Lipid based carriers nanoformulations include liposomes; Drug conjugates include antibody-drug conjugates and polymer-drug conjugates; Polymeric carriers include nanoparticle albumin bound technology, polymeric micelles and polymeric nanoparticles [77,89].

The relevance of heterocyclic compounds in nanoparticles conjugated based therapy is highlighted by the increasing number of FDA approved nanoconjugated therapies, and by the high number of ongoing clinical trials (Figure 20) [77,89]. Interestingly, the fact there is a number of major nanoformulations in several clinical trial stages should be noted (Figure 20). For instance, among the nanoformulations using heterocyclic compounds, 33% and 32% of clinical trials consist in the conjugation of heterocyclic compounds with antibodies or liposomes, respectively. However, almost 50% of the nanoformulations using heterocyclic compounds approved by FDA were based on liposomes.

Doxorubicin is one of the most used compounds in nanoformulations for cancer therapy. Indeed, three different products (Doxil^®^/Caelyx^®^ from Johnson & Johnson, Lipo-Dox^®^ from Taiwan Liposome and Myocet^®^ from Cephalon) were approved using liposomes as nanocarriers of doxorubicin for treatment of Kaposi’s sarcoma and breast and ovarian cancer [102,103,104]. Indeed, encapsulation of doxorubicin in liposomes seems to maintain efficacy against the above referred tumors, with improved pharmacokinetics that include longer circulating half-life and slow plasma clearance of the heterocyclic compound [102,103,105]. One of the advantages in using these nanoformulations seems to be a reduced cardiotoxicity and neutropenia, registered in the Myocet clinical trial [104]. Currently, Myocet^®^ is used in the first line treatment of metastatic breast cancer in conjugation with cyclophosphamide [106], while Doxil^®^, Caelix^®^ or Lipo-Dox® are mainly indicated as first-line monotherapy of patients with metastatic breast cancer, advanced ovarian cancer, and as second-line treatment of AIDS-related Kaposi’s sarcoma [103,107].

Moreover, several nanoformulations using Paclitaxel were also approved (e.g., Abraxane® from Abraxis/Celgene, nanoparticle albumin bound) and are in the phase III of clinical trials [77]. Abraxane is currently indicated for metastatic breast cancer treatment after failure of combination chemotherapy, and as first-line treatment of non-small cell lung cancer and advanced pancreatic cancer in combination with gemcitabine. However, several adverse effects were still encountered with an incidence higher than 5%, including anemia, neutropenia and thrombocytopenia.

Other promising nanoformulations that reached phase III include Irinotecan conjugated with PEG (NKTR-102 from Nektar) [108] or Cytarabine/daunorubicin in liposomes (CPX-351 from Celator) [109]. Despite that drug-related toxicity is still registered in phase II trials, 29% of the patients with metastatic breast cancer in clinical trial phase II registered objective response for Irinotecan pegol (NKTR-102) [108], and 21% of relapsed and refractory acute myeloid leukemia patients in clinical trial II registered complete response to CPX-351 [109].

Moreover, several nanoformulations using Paclitaxel were also approved (e.g., Abraxane^®^ from Abraxis/Celgene, nanoparticle albumin bound) and are in the phase III of clinical trials [61]. Other promising nanoformulations that reached phase III include Irinotecan conjugated with PEG (NKTR-102 from Nektar) [108], Cytarabine/daunorubicin in liposomes (CPX-351 from Celator) [109] or Vincristin in liposomes (Onco-TCS from Inex/Enzon) [110].

Despite the promising effects of these nanoformulations, clinicians still struggle with cancer recurrence and metastasis management. Currently, an effort is being made to design and develop multifunctional nanosystems capable of performing diagnostics and therapeutics in real time (nanotheranostics). The use of gold nanoparticles has been successfully proven *in vitro* to this end [74,111,112].

### 2.3. Fundamental Aspects of Nanoformulation Design and in Vivo Interactions

#### 2.3.1. Nanomaterial–Cell Interactions and Cell Uptake

Either considering the application of nanomedicine’s toolbox for heterocyclic compound vectorization in cancer, or any other application of nanoscale resources suitable for biomedicine (e.g., targeted therapy, molecular diagnostics, theranostics), one has to keep in mind that the theoretical and experimental research that has taken place over the last decades related to nanoscience and nanobiotechnology has raised several biological questions intimately connected with public health issues. Perhaps at the forefront of these biological questions is how such nanoparticulate formulations interact with the human body based on the role of the particle size, surface area, material composition, shape, surface chemistry and overall charge [113,114]. All of these inherent characteristics will eventually determine how the nanoparticulate formulations cross barriers, interact with biological fluids, and how and where these will interfere with protein interactions and other fundamental molecular players that comprehend natural entities in biological systems. These will influence the delivery efficiency of the drug payload into the target cell/tissue and the overall effect of the nanoformulation itself (benign or toxic). Similarly with many toxicological agents, several nanoparticle systems are prone to interact adversely with cellular metabolism; however, some reports are inconsistent or conflicting. For instance, while several authors report the biocompatibility of gold nanoparticles [115,116], others seem to contradict these findings [117,118]. This situation is also evident for other types of nanoparticles such as carbon nanotubes, for instance [119,120]. 

Nonetheless, considering the full spectrum of physicochemical properties that a single nanoparticle might encompass in its formulation, it is not yet known exactly how these fully interact with cell/tissue or, for that matter with the plethora of molecular factors at work at a the cellular scale. It is suggested though, that nanoparticles when in contact with physiological conditions, due to their small size and chemistry, interact with opsonins and may act like haptens by altering proteins at the surface of different cells, facilitating their uptake by mammalian cells. In particular, authors have attempted to correlate the size dependence of nanoparticles and uptake kinetics into mammalian cells. Several reports state that maximum uptake by cells occurs at a (gold)nanoparticle size of around 50 nm [121,122,123], while other studies have reported that this effect might equally be dependent on the composition of the nanoparticle itself. For instance, a study conducted using polystyrene nanoparticles to determine their uptake efficiency in Caco-2, a human colon adenocarcinoma cell line, contradicted the idea that 50 nm is the ideal size for internalization. The authors concluded that in fact the efficiency of internalization for 50 nm particles was the lowest out of a range of different sizes. Interestingly, here nanoparticles two times larger were significantly more internalized by cells [124]. Following the same rationale basis, it is easy to understand that depending on the different moieties functionalized onto the nanoparticles surface, the uptake and intracellular fate of such nanoformulations will be conditioned. Literature reports demonstrate that endosomal route of uptake can be conditioned by surface modification of nanoparticles with different biological moieties such as penetrating peptides [125]. Moreover, the specific moieties functionalized onto a nanocarrier to target a subset of cells towards a select route may actually have different uptake mechanisms as compared to the moiety alone [126].

The advent of nanoparticles as drug carriers has led without doubt to an increasing need to understand nanoparticle endocytosis mechanisms. The main energy dependent uptake processes involves phagocytosis, pinocytosis, and caveolae-dependent or clathrin-mediated endocytosis, and the activation of such methods is not yet clear although it is known to be dependent on the overall shape, size, and physicochemical properties [113].

#### 2.3.2. Nanoparticle Size and Shape

The success of an effective drug delivery system is size and shape dependent. Both characteristics influence blood circulation time and biodistribution when administrated to a patient [127,128].

Shaping nanoparticles may prove to be a useful tool in cancer targeting. When comparing spherical nanoparticles to oblate-shaped ones, the latter are subjected to torques resulting in lateral drift due to tumbling and rotation. This phenomenon directs particles towards the blood vessel walls [127,129].

Nanoparticles bigger than a few nanometers, e.g., 7 nm, and smaller than 400 nm are more likely to accumulate in tumors due to the leakiness of tumor vessels caused by fenestrations between defective endothelial cells [128,130].

#### 2.3.3. Natural Barriers

Relatively large nanoparticles, in order to reach tumor cells, must pass through a highly interconnected network of collagen fibers. This barrier may significantly reduce the amount of nanoparticles reaching the tumor, by nonspecific interactions or simply by hindering nanoparticle movement, thus, leading to the nanoparticles being concentrated in the vicinity of blood vessels that they leaked from [126,129,130,131].

To be effective, nanoparticles must avoid recognition by the reticulo-endothelial system (RES) composed of macrophages. Generally, particles larger than 100 nm are rapidly eliminated from the circulation by the RES [128].

In addition, macrophages recognize nanoparticles as foreign bodies which results in a rapid uptake and elimination [128,132]. In order to evade these specialized cells of the RES, and increase the circulation time, nanoparticles may be coated with several polymer moieties to avoid opsonization and phagocytosis. Among these polymer moieties, PEG is the most commonly used to this end [128,132].

#### 2.3.4. Drug Release Rate

The loading ratio of drug into the nanoparticle may lead to challenges in achieving the required dose to be delivered into the tumor. Lower ratios of drug loading lead to insufficient accumulation in the tumor. Another limiting factor is the fast release rate in circulation.

Conjugates of nanoparticles with drugs may be synthetized by either covalent binding of the drug to the nanoparticle surface, or, entrapment by either non-covalent or covalent bound. In reality, various chemical bonds (amide, ester, azide, *etc*.) are used to design nanoconjugates. The release of drugs from nanoparticles depends on the nature of these chemical bonds. The release may be dependent on either pH (usually acidic pH), temperature or on enzymatic cleavage. The nature of the chemical bound will also determine the release rate. In order for the nanocarrier to delivery its drug content into the tumor, the chemical bond should be stable upon blood circulation, preventing its rapid release and promoting the therapeutic effect in the tumor site [128,133]. A rapid release rate may produce results similar to free drugs in terms of biodistribution and toxicity [128], however, this strategy limits the interaction of nanoparticles with the tumor cell membrane and decreases uptake by tumor cells [128].

### 2.4. Challenges Still to Overcome

Nanomedicine, in the last couple of years, has emerged as one of the most promising and advanced technological fields in cancer treatment. Each year, thousands of publications reinforce this predicament. However, despite the thousands of publications, suggesting new nanosytems for cancer therapy, only a handful have entered clinical trials, and even less are approved for clinical use. Several challenges still have to be overcome, such as physicochemical characterization, safety concerns, regulatory issues and manufacturing issues [77]. 

The physicochemical features of a nanocarrier comprise of its structure, size, composition, surface, and charge. Due to variability in these parameters, it is difficult to accurately characterize the nanodevices administered [77,134,135]. One of the most important aspects is polydispersity, which measures the heterogeneity of nanoparticles regarding size, shape or mass. A small variation in the polydispersity index may cause changes in other factors, such as toxicity. So, a wide variety of methods should be used on a batch-to-batch basis, to accurately characterize the nanodevices. Thus, all methods should be performed under conditions mimicking physiological conditions, and interaction between physiological fluids should be well understood as a means to guarantee the optimal functionality of the nanodevices. Stability and storage aspects should also be well understood and characterized [77].

The rapid growth of nanoparticles in medicine makes it necessary to address toxicity of this nanodevices in human health. Toxicity assays used for nanodevices are the same used for drugs, possibly leading to an inadequate assessment. As toxicity of nanodevices is dependent on several factors, such as shape, size, surface area, surface charge or porosity, it is necessary to encourage the development of complementary toxicity assessments [136]. Despite the possibility of an inadequate use of toxicity assays, acute toxicity of nanodevices is a well-studied factor, however, data concerning chronic toxicity is lacking [77]. 

Other factors lacking consist of a tight legislation and guidelines. FDA and European Medicines Agency have yet not implemented the previously mentioned guidelines concerning nanomaterials. However, FDA recently outlined in publication their approach to the regulation of nanomaterials [137]. Without valid guidelines, approvals and regulatory decisions of nanomaterials would be based on individual assessment of benefit-risk resulting in a bottleneck [138]. Both FDA and Nanotechnology Characterization Laboratory (NCL), in a joint effort, are gathering information to solve the problems mentioned above. More than 40 protocols have been established to address nanoparticles’ physicochemical characteristics, both *in vitro* and *in vivo* toxicity, and biodistribution and clearance of nanoparticles using animal models [74].

## 3. Conclusions

Naturally occurring heterocycles seem to play an important role in biochemical reactions in cells’ metabolism. Their reactivity with cells and tissues makes the regulation of these molecules so tightly controlled that as a consequence any disturbance may be associated with pathological conditions. Therefore, the use of synthetic cyclic compounds as anticancer drugs tries to mimic natural ligands and substrates in order to disturb the delicate balance in cells. Heterocyclic compounds or heterocyclic fragments also play an important role in improving pharmacokinetics and pharmacodynamics properties of anticancer drugs by enhancing lipophilicity, polarity or other physicochemical features. Hence, heterocycles play an important role in current drug design as they are present in the majority of marketed drugs. Only in 2015, about 30% of FDA-approved anticancer drugs have one or more cyclic rings containing nitrogen or oxygen. A correlation between heterocycle fragments’ structure and potential families of targeted molecules seems to not be evidenced by any literature addressed. However, mechanisms of action of these compounds are being established and pass through interactions with major biomolecules or by intervening in metabolic pathways. Although heterocycles or heterocycle like compounds improve pharmacokinetic and pharmacodynamics, it still faces many challenges such as lack of specific targeting. Therefore, it is imperative to search for a method to overcome these issues.

Nanovectorization strategies are a promising solution with benefits that range from enhanced permeability and retention effect, and passive targeting for better biocompatibility. It is also possible to upgrade active targeting as means of improving cancer cells’ selectivity. There are several options to nanovectorize drugs such as polymeric nanoparticles, albumin bound nanoparticles, metallic nanoparticles and dendrimers, however, liposomes are the major class with ongoing clinical trials. Although there are thousands of publications regarding nanodevices conjugated with anticancer drugs, few nanoconjugates have entered into clinical trials and fewer are approved for human use. Many challenges still need to be answered such as an optimal and reproducible physicochemical characterization of nanomaterials for an industrialized vectorization of compounds, safety concerns, and regulatory and manufacturing issues in order to achieve a rapidly efficacious treatment for cancer patients.

## References

[1] IUPAC Gold Book—Heterocyclic Compounds. http://goldbook.iupac.org/H02798.html.

[2] Gomtsyan A. (2012). Heterocycles in drugs and drug discovery. Chem. Heterocycl. Compd..

[3] Dua R., Shrivastava S., Sonwane S.K., Srivastava S.K. (2011). Pharmacological Significance of Synthetic Heterocycles Scaffold : A Review. Adv. Biol. Res. (Rennes)..

[4] Eicher T., Hauptmann S., Speicher A. (2012). The Structure of Heterocyclic Compounds. The Chemistry of Heterocycles: Structure, Reactions, Synthesis, and Applications.

[5] Broughton H.B., Watson I.A. (2004). Selection of heterocycles for drug design. J. Mol. Graph. Model..

[6] El-salam N.M.A., Mostafa M.S., Ahmed G.A., Alothman O.Y. (2013). Synthesis and Antimicrobial Activities of Some New Heterocyclic Compounds Based on 6-Chloropyridazine-3 (2*H*) -thione. J. Chem..

[7] Azab M.E., Youssef M.M., El-Bordany E.A. (2013). Synthesis and antibacterial evaluation of novel heterocyclic compounds containing a sulfonamido moiety. Molecules.

[8] Salem M.S., Sakr S.I., El-Senousy W.M., Madkour H.M.F. (2013). Synthesis, antibacterial, and antiviral evaluation of new heterocycles containing the pyridine moiety. Arch. Pharm. (Weinheim)..

[9] Cao X., Sun Z., Cao Y., Wang R., Cai T., Chu W., Hu W., Yang Y. (2014). Design, Synthesis, and Structure—Activity Relationship Studies of Novel Fused Heterocycles-Linked Triazoles with Good Activity and Water Solubility. J. Med. Chem..

[10] El-Sawy E.R., Ebaid M.S., Abo-Salem H.M., Al-Sehemi A.G., Mandour A.H. (2013). Synthesis, anti-inflammatory, analgesic and anticonvulsant activities of some new 4,6-dimethoxy-5-(heterocycles)benzofuran starting from naturally occurring visnagin. Arab. J. Chem..

[11] Chen Y., Yu K., Tan N.Y., Qiu R.H., Liu W., Luo N.L., Tong L., Au C.T., Luo Z.Q., Yin S.F. (2014). Synthesis, characterization and anti-proliferative activity of heterocyclic hypervalent organoantimony compounds. Eur. J. Med. Chem..

[12] El-Sawy E.R., Mandour A.H., El-Hallouty S.M., Shaker K.H., Abo-Salem H.M. (2013). Synthesis, antimicrobial and anticancer activities of some new N-methylsulphonyl and N-benzenesulphonyl-3-indolyl heterocycles. 1st Cancer Update. Arab. J. Chem..

[13] Mabkhot Y.N., Barakat A., Al-Majid A.M., Alshahrani S., Yousuf S., Choudhary M.I. (2013). Synthesis, reactions and biological activity of some new bis-heterocyclic ring compounds containing sulphur atom. Chem. Cent. J..

[14] Alvárez-Builla J., Barluenga J. (2011). Heterocyclic Compounds: An Introduction. Mod. Heterocycl. Chem..

[15] Top Prescription Drugs by U.S. Sales 2014|Statistic. http://www.statista.com/statistics/258010/top-branded-drugs-based-on-retail-sales-in-the-us/.

[16] Peer D., Karp J.M., Hong S., Farokhzad O.C., Margalit R., Langer R. (2007). Nanocarriers as an emerging platform for cancer therapy. Nat. Nanotechnol..

[17] Hambley T.W., Hait W.N. (2009). Is anticancer drug development heading in the right direction?. Cancer Res..

[18] Martins P., Rosa D., Fernandes A.R., Baptista P.V. (2014). Nanoparticle Drug Delivery Systems : Recent Patents and Applications in Nanomedicine. Recent Pat. Nanomed..

[19] Conde J., Ambrosone A., Sanz V., Hernandez Y., Marchesano V., Tian F., Child H., Berry C.C., Ibarra M.R., Baptista P.V., Tortiglione C., Fuente J.M. (2012). De Design of Multifunctional Gold Nanoparticles for *in Vitro* and *in Vivo* Gene Silencing. ACS Nano.

[20] Conde J., Doria G., Baptista P. (2012). Noble metal nanoparticles applications in cancer. J. Drug Deliv..

[21] Market Opportunities in Nanotechnology Drug Delivery. http://www.cientifica.com/research/white-papers/market-opportunities-in-nanotechnology-drug-delivery/.

[22] Research, C. for D.E. and New Drugs at FDA: CDER’s New Molecular Entities and New Therapeutic Biological Products. http://www.fda.gov/Drugs/DevelopmentApprovalProcess/DrugInnovation/default.htm.

[23] Click2Drug—Encyclopedia—Chemical Compounds—Most Frequent Rings in FDA Approved Drugs. http://www.click2drug.org/encyclopedia/chemistry/fda-based-rings.html.

[24] Vitaku E., Smith D.T., Njardarson J.T. (2014). Analysis of the Structural Diversity, Substitution Patterns, and Frequency of Nitrogen Heterocycles among U.S. FDA Approved Pharmaceuticals. J. Med. Chem..

[25] Ali N.A.S., Dar B.A., Pradhan V., Farooqui M. (2013). Chemistry and biology of indoles and indazoles: a mini-review. Mini Rev. Med. Chem..

[26] Kaushik N.K., Kaushik N., Attri P., Kumar N., Kim C.H., Verma A.K., Choi E.H. (2013). Biomedical importance of indoles. Molecules.

[27] Sherer C., Snape T.J. (2015). Heterocyclic scaffolds as promising anticancer agents against tumours of the central nervous system: Exploring the scope of indole and carbazole derivatives. Eur. J. Med. Chem..

[28] Brancale A., Silvestri R. (2007). Indole, a core nucleus for potent inhibitors of tubulin polymerization. Med. Res. Rev..

[29] Kumar S., Mehndiratta S., Nepali K., Gupta M.K., Koul S., Sharma P.R., Saxena A.K., Dhar K.L. (2013). Novel indole-bearing combretastatin analogues as tubulin polymerization inhibitors. Org. Med. Chem. Lett..

[30] Huang S.M., Hsu P.C., Chen M.Y., Li W.S., More S.V., Lu K.T., Wang Y.C. (2012). The novel indole compound SK228 induces apoptosis and FAK/Paxillin disruption in tumor cell lines and inhibits growth of tumor graft in the nude mouse. Int. J. Cancer.

[31] Verma A., Joshi S., Singh D. (2013). Imidazole: Having versatile biological activities. J. Chem..

[32] Sharma G.V.M., Ramesh A., Singh A., Srikanth G., Jayaram V., Duscharla D., Jun J.H., Ummanni R., Malhotra S.V. (2014). Imidazole derivatives show anticancer potential by inducing apoptosis and cellular senescence. Med. Chem. Commun..

[33] Hou J., Zhao W., Huang Z.-N., Yang S.-M., Wang L.-J., Jiang Y., Zhou Z.-S., Zheng M.-Y., Jiang J.-L., Li S.-H. (2014). Evaluation of Novel N -(piperidine-4-yl)benzamide Derivatives as Potential Cell Cycle Inhibitors in HepG2 Cells. Chem. Biol. Drug Des..

[34] Khan I., Ibrar A., Abbas N. (2013). Triazolothiadiazoles and triazolothiadiazines-Biologically attractive scaffolds. Eur. J. Med. Chem..

[35] Husain A., Rashid M., Shaharyar M., Siddiqui A.A., Mishra R. (2013). Benzimidazole clubbed with triazolo-thiadiazoles and triazolo-thiadiazines: New anticancer agents. Eur. J. Med. Chem..

[36] Husain A., Rashid M., Mishra R., Parveen S., Shin D.S., Kumar D. (2012). Benzimidazole bearing oxadiazole and triazolo-thiadiazoles nucleus: Design and synthesis as anticancer agents. Bioorg. Med. Chem. Lett..

[37] Kamel M.M., Megally Abdo N.Y. (2014). Synthesis of novel 1,2,4-triazoles, triazolothiadiazines and triazolothiadiazoles as potential anticancer agents. Eur. J. Med. Chem..

[38] Mekhail T.M., Markman M. (2002). Paclitaxel in cancer therapy. Expert Opin. Pharmacother..

[39] Vrignaud P., Sémiond D., Lejeune P., Bouchard H., Calvet L., Combeau C., Riou J.F., Commerçon A., Lavelle F., Bissery M.C. (2013). Preclinical antitumor activity of cabazitaxel, a semisynthetic taxane active in taxane-resistant tumors. Clin. Cancer Res..

[40] Devriese L.A., Mergui-Roelvink M., Wanders J., Jenner A., Edwards G., Reyderman L., Copalu W., Peng F., Marchetti S., Beijnen J.H. (2013). Eribulin mesylate pharmacokinetics in patients with solid tumors receiving repeated oral ketoconazole. Investig. New Drugs.

[41] Yadagiri B., Holagunda U.D., Bantu R., Nagarapu L., Kumar C.G., Pombala S., Sridhar B. (2014). Synthesis of novel building blocks of benzosuberone bearing coumarin moieties and their evaluation as potential anticancer agents. Eur. J. Med. Chem..

[42] Kontogiorgis C., Detsi A., Hadjipavlou-litina D. (2012). Coumarin-Based Drugs : A Patent Review (2008–present ). Expert Opin. Ther. Pat..

[43] Kaur M., Kohli S., Sandhu S., Bansal Y., Bansal G. (2015). Coumarin: A Promising Scaffold for Anticancer Agents. Anticancer Agents Med. Chem..

[44] Chen X., Zhou J.H., Huang Q., Lu L., Min W. (2014). Novel Action and Mechanism of Auranofin in Inhibition of Vascular Endothelial Growth Factor Receptor-3-Dependent Lymphangiogenesis|BenthamScience. Anti-Cancer Agents.

[45] Liu N., Li X., Huang H., Zhao C., Liao S., Yang C., Liu S., Song W., Lu X., Lan X. (2014). Clinically used antirheumatic agent auranofin is a proteasomal deubiquitinase inhibitor and inhibits tumor growth. Oncotarget.

[46] Park S.-H., Lee J., Berek J., Hu M. (2014). Auranofin displays anticancer activity against ovarian cancer cells through FOXO3 activation independent of p53. Int. J. Oncol..

[47] Murti Y., Mishra P. (2014). Synthesis and evaluation of flavanones as anticancer agents. Indian J. Pharm. Sci..

[48] Khanam H. (2014). Shamsuzzaman Bioactive Benzofuran derivatives: A review. Eur. J. Med. Chem..

[49] Choi M., Jo H., Park H.-J., Sateesh Kumar A., Lee J., Yun J., Kim Y., Han S., Jung J.-K., Cho J. (2015). Design, synthesis, and biological evaluation of benzofuran- and 2,3-dihydrobenzofuran-2-carboxylic acid *N*-(substituted)phenylamide derivatives as anticancer agents and inhibitors of NF-κB. Bioorg. Med. Chem. Lett..

[50] Rodrigues F.A.R., Bomfim I.D.S., Cavalcanti B.C., Pessoa C., Goncalves R.S.B., Wardell J.L., Wardell S.M.S.V., de Souza M.V.N. (2014). Mefloquine-Oxazolidine Derivatives: A New Class of Anticancer Agents. Chem. Biol. Drug Des..

[51] Andrade S.F., Teixeira C.S., Ramos J.P., Lopes M.S., Pádua R.M., Oliveira M.C., Souza-Fagundes E.M., Alves R.J. (2014). Synthesis of a novel series of 2,3,4-trisubstituted oxazolidines designed by isosteric replacement or rigidification of the structure and cytotoxic evaluation. Med. Chem. Commun..

[52] Khatik G.L., Kaur J., Kumar V., Tikoo K., Nair V.A. (2012). 1,2,4-Oxadiazoles: A new class of anti-prostate cancer agents. Bioorg. Med. Chem. Lett..

[53] Valente S., Trisciuoglio D., de Luca T., Nebbioso A., Labella D., Lenoci A., Bigogno C., Dondio G., Miceli M., Brosch G. (2014). 1,3,4-Oxadiazole-containing histone deacetylase inhibitors: Anticancer activities in cancer cells. J. Med. Chem..

[54] García-Valverde M., Torroba T. (2005). Special Issue: Sulfur-Nitrogen Heterocycles. Molecules.

[55] Marcos C.F., Polo C., Rakitin O.A., Rees C.W., Torroba T. (1997). From Hiinig’s Base to Bis([l,2]dithiolo)-[1,4]thiazines in One Pot: The Fast Route to Highly Sulfurated Heterocycles. Angew. Chem. Int. Ed. Engl..

[56] Makki M.S.T., Abdel-rahman R.M., El-Shahawi M.S. (2011). Synthesis of New Bioactive Sulfur Compounds Bearing Heterocyclic Moiety and Their Analytical Applications. Int. J. Chem..

[57] Toohey J., Cooper A. (2014). Thiosulfoxide (Sulfane) Sulfur: New Chemistry and New Regulatory Roles in Biology. Molecules.

[58] Said M., Elshihawy H. (2014). Synthesis, anticancer activity and structure-activity relationship of some anticancer agents based on Cyclopenta (*b*) thiophene scaffold. Pak. J. Pharm. Sci..

[59] Ghorab M.M., Bashandy M.S., Alsaid M.S. (2014). Novel thiophene derivatives with sulfonamide, isoxazole, benzothiazole, quinoline and anthracene moieties as potential anticancer agents. Acta Pharm..

[60] Carter J.S., Kramer S., Talley J.J., Penning T., Collins P., Graneto M.J., Seibert K., Koboldt C.M., Masferrer J., Zweifel B. (1999). Synthesis and activity of sulfonamide-substituted 4,5-diaryl thiazoles as selective cyclooxygenase-2 inhibitors. Bioorg. Med. Chem. Lett..

[61] Rudolph J., Theis H., Hanke R., Endermann R., Johannsen L., Geschke F. (2001). seco-Cyclothialidines: new concise synthesis, inhibitory activity toward bacterial and human DNA topoisomerases, and antibacterial properties. J. Med. Chem..

[62] Liu X.H., Shi Y.X., Ma Y., Zhang C.Y., Dong W.L., Pan L., Wang B.L., Li B.J., Li Z.M. (2009). Synthesis, antifungal activities and 3D-QSAR study of *N*-(5-substituted-1,3,4-thiadiazol-2-yl)cyclopropanecarboxamides. Eur. J. Med. Chem..

[63] Bell F.W., Cantrell A.S., Högberg M., Jaskunas S.R., Johansson N.G., Jordan C.L., Kinnick M.D., Lind P., Morin J.M., Noréen R. (1995). Phenethylthiazolethiourea (PETT) compounds, a new class of HIV-1 reverse transcriptase inhibitors. 1. Synthesis and basic structure-activity relationship studies of PETT analogs. J. Med. Chem..

[64] Laczkowski K.Z.K.M., Switalska M., Wietrzyk J., Baranowska A.L., Berta F., Paneth A., Plech T. (2014). Synthesis and *in Vitro* Antiproliferative Activity of Thiazole-Based Nitrogen Mustards: The Hydrogen Bonding Interaction between Model Systems and Nucleobases. Anti-Cancer Agents.

[65] Penthala N.R., Sonar V.N., Horn J., Leggas M., Yadlapalli J.S.K.B., Crooks P.A. (2012). Synthesis and evaluation of a series of benzothiophene acrylonitrile analogs as anticancer agents. MedChemComm.

[66] Molica S. (2013). The emerging role of ibrutinib in the treatment of chronic lymphocytic leukemia. Expert Rev. Hematol..

[67] Fiorcari S., Brown W.S., McIntyre B.W., Estrov Z., Maffei R., O’Brien S., Sivina M., Hoellenriegel J., Wierda W.G., Keating M.J. (2013). The PI3-kinase delta inhibitor idelalisib (GS-1101) targets integrin-mediated adhesion of chronic lymphocytic leukemia (CLL) cell to endothelial and marrow stromal cells. PLoS ONE.

[68] Fact Sheets by Population. http://globocan.iarc.fr/Pages/fact_sheets_population.aspx.

[69] Cadoo K.A., Gucalp A., Traina T.A. (2014). Palbociclib: An evidence-based review of its potential in the treatment of breast cancer. Dove Press.

[70] Home—PubChem Compound—NCBI. http://www.ncbi.nlm.nih.gov/pccompound.

[71] Noori H.R., Spanagel R. (2013). *In silico* pharmacology: Drug design and discovery’s gate to the future. Silico Pharmacol..

[72] Meyer E.F., Swanson S.M., Williams J.A. (2000). Molecular modelling and drug design. Pharmacol. Ther..

[73] Chaniyara R., Tala S., Chen C.W., Lee P.C., Kakadiya R., Dong H., Marvania B., Chen C.H., Chou T.C., Lee T.C. (2012). Synthesis and antitumor evaluation of novel Benzo[*d*]pyrrolo[2,1-*b*]thiazole derivatives. Eur. J. Med. Chem..

[74] Baptista P., Fernandes A., Figueiredo S., Vinhas R., Cordeiro M., Carlos F., Mendo S. (2015). Gold nanoparticle-based theranostics: Disease diagnostics and treatment using a single nanomaterial. Nanobiosen. Dis. Diagn..

[75] Xu X., Ho W., Zhang X., Bertrand N., Farokhzad O. (2015). Cancer nanomedicine: From targeted delivery to combination therapy. Trends Mol. Med..

[76] Sagnella S.M., McCarroll J.A., Kavallaris M. (2014). Drug delivery: Beyond active tumour targeting. Nanomed. Nanotechnol. Biol. Med..

[77] Wicki A., Witzigmann D., Balasubramanian V., Huwyler J. (2015). Nanomedicine in cancer therapy: Challenges, opportunities, and clinical applications. J. Control. Release.

[78] Estanqueiro M., Amaral M.H., Conceição J., Sousa Lobo J.M. (2015). Nanotechnological carriers for cancer chemotherapy: The state of the art. Colloids Surf. B Biointerfaces.

[79] Zhang L., Gu F.X., Chan J.M., Wang A.Z., Langer R.S., Farokhzad O.C. (2008). Nanoparticles in Medicine : Therapeutic Applications and Developments. Clin. Pharmacol. Ther..

[80] Allen T.M., Cullis P.R. (2013). Liposomal drug delivery systems: From concept to clinical applications. Adv. Drug Deliv. Rev..

[81] Pérez-Herrero E., Fernández-Medarde A. (2015). Advanced targeted therapies in cancer: Drug nanocarriers, the future of chemotherapy. Eur. J. Pharm. Biopharm..

[82] Wang A.Z., Langer R., Farokhzad O.C. (2012). Nanoparticle delivery of cancer drugs. Annu. Rev. Med..

[83] Szebeni J. (2005). Complement activation-related pseudoallergy: A new class of drug-induced acute immune toxicity. Toxicology.

[84] Parhi P., Mohanty C., Sahoo S.K. (2012). Nanotechnology-based combinational drug delivery: An emerging approach for cancer therapy. Drug Discov. Today.

[85] Nazir S., Hussain T., Ayub A., Rashid U., MacRobert A.J. (2014). Nanomaterials in combating cancer: Therapeutic applications and developments. Nanomedicine.

[86] Gillies E.R., Fréchet J.M.J. (2005). Dendrimers and dendritic polymers in drug delivery. Drug Discov. Today.

[87] Kesharwani P., Iyer A.K. (2014). Recent advances in dendrimer-based nanovectors for tumor-targeted drug and gene delivery. Drug Discov. Today.

[88] Sanna V., Pala N., Sechi M. (2014). Targeted therapy using nanotechnology: Focus on cancer. Int. J. Nanomed..

[89] Chari R.V.J., Miller M.L., Widdison W.C. (2014). Antibody-drug conjugates: An emerging concept in cancer therapy. Angew. Chem. Int. Ed..

[90] Elzoghby A.O., Samy W.M., Elgindy N.A. (2012). Albumin-based nanoparticles as potential controlled release drug delivery systems. J. Control. Release.

[91] Cabral R.M., Baptista P.V. (2013). The Chemistry and Biology of Gold Nanoparticle-Mediated Photothermal Therapy: Promises and Challenges. Nano Life.

[92] Peralta D.V., Heidari Z., Dash S., Tarr M.A. (2015). Hybrid Paclitaxel and Gold Nanorod-Loaded Human Serum Albumin Nanoparticles for Simultaneous Chemotherapeutic and Photothermal Therapy on 4T1 Breast Cancer Cells. ACS Appl. Mater. Interfaces.

[93] Zolot R.S., Basu S., Million R.P. (2013). Antibody–drug conjugates. Nat. Rev. Drug Discov..

[94] Teicher B.A. (2014). Antibody drug conjugates. Curr. Opin. Oncol..

[95] Panowski S., Bhakta S., Raab H., Polakis P., Junutula J.R. (2014). Site-specific antibody drug conjugates for cancer therapy. MAbs.

[96] Dhillon S. (2015). Dinutuximab: First Global Approval. Drugs.

[97] Falchook G. (2015). Nivolumab: Another weapon in the growing immunotherapy arsenal. Lancet Oncol..

[98] Robert C., Long G.V., Brady B., Dutriaux C., Maio M., Mortier L., Hassel J.C., Rutkowski P., McNeil C., Kalinka-Warzocha E. (2015). Nivolumab in Previously Untreated Melanoma without BRAF Mutation. N. Engl. J. Med..

[99] Flygare J.A., Pillow T.H., Aristoff P. (2013). Antibody-Drug Conjugates for the Treatment of Cancer. Chem. Biol. Drug Des..

[100] Ornes S. (2013). Antibody–drug conjugates. Proc. Natl. Acad. Sci. USA.

[101] Casi G., Neri D. (2012). Antibody-drug conjugates: Basic concepts, examples and future perspectives. J. Control. Release.

[102] Muggia F.M., Hainsworth J.D., Jeffers S., Miller P., Groshen S., Tan M., Roman L., Uziely B., Muderspach L., Garcia A. (1997). Phase II study of liposomal doxorubicin in refractory ovarian cancer: Antitumor activity and toxicity modification by liposomal encapsulation. J. Clin. Oncol..

[103] Khemapech N., Oranratanaphan S., Termrungruanglert W., Lertkhachonsuk R., Vasurattana A. (2013). Salvage chemotherapy in recurrent platinum-resistant or refractory epithelial ovarian cancer with Carboplatin and distearoylphosphatidylcholine pegylated liposomal Doxorubicin (lipo-dox^®^). Asian Pac. J. Cancer Prev..

[104] Batist G., Ramakrishnan G., Rao C.S., Chandrasekharan A., Gutheil J., Guthrie T., Shah P., Khojasteh A., Nair M.K., Hoelzer K. (2001). Reduced cardiotoxicity and preserved antitumor efficacy of liposome-encapsulated doxorubicin and cyclophosphamide compared with conventional doxorubicin and cyclophosphamide in a randomized, multicenter trial of metastatic breast cancer. J. Clin. Oncol..

[105] Tejada-Berges T., Granai C.O., Gordinier M., Gajewski W. (2002). Caelyx/Doxil for the treatment of metastatic ovarian and breast cancer. Expert Rev. Anticancer Ther..

[106] European Medicines Agency—Find Medicine—Myocet. http://www.ema.europa.eu/ema/index.jsp?curl=pages/medicines/human/medicines/000297/human_med_000916.jsp&mid=WC0b01ac058001d124.

[107] European Medicines Agency—Find medicine—Caelyx. http://www.ema.europa.eu/ema/index.jsp?curl=pages/medicines/human/medicines/000089/human_med_000683.jsp&mid=WC0b01ac058001d124.

[108] Awada A., Garcia A.A., Chan S., Jerusalem G.H.M., Coleman R.E., Huizing M.T., Mehdi A., O’Reilly S.M., Hamm J.T., Barrett-Lee P.J. (2013). Two schedules of etirinotecan pegol (NKTR-102) in patients with previously treated metastatic breast cancer: A randomised phase 2 study. Lancet Oncol..

[109] Feldman E.J., Lancet J.E., Kolitz J.E., Ritchie E.K., Roboz G.J., List A.F., Allen S.L., Asatiani E., Mayer L.D., Swenson C. (2011). First-in-man study of CPX-351: A liposomal carrier containing cytarabine and daunorubicin in a fixed 5:1 molar ratio for the treatment of relapsed and refractory acute myeloid leukemia. J. Clin. Oncol..

[110] Sarris A.H., Hagemeister F., Romaguera J., Rodriguez M.A., McLaughlin P., Tsimberidou A.M., Medeiros L.J., Samuels B., Pate O. (2000). Liposomal vincristine in relapsed non-Hodgkin’s lymphomas: Early results of an ongoing phase II trial. Ann. Oncol..

[111] Conde J., Larguinho M., Cordeiro A., Raposo L.R., Costa P.M., Santos S., Diniz M.S., Fernandes A.R., Baptista P. (2014). V Gold-nanobeacons for gene therapy: Evaluation of genotoxicity, cell toxicity and proteome profiling analysis. Nanotoxicology.

[112] R. Fernandes A., Viana Baptista P. (2013). Nanotechnology for Cancer Diagnostics and Therapy – An Update on Novel Molecular Players. Curr. Cancer Ther. Rev..

[113] Zhao F., Zhao Y., Liu Y., Chang X., Chen C., Zhao Y. (2011). Cellular uptake, intracellular trafficking, and cytotoxicity of nanomaterials. Small.

[114] Iversen T.G., Skotland T., Sandvig K. (2011). Endocytosis and intracellular transport of nanoparticles: Present knowledge and need for future studies. Nano Today.

[115] Shukla R., Bansal V., Chaudhary M., Basu A., Bhonde R.R., Sastry M. (2005). Biocompatibility of gold nanoparticles and their endocytotic fate inside the cellular compartment: A microscopic overview. Langmuir.

[116] Murugan M., Anthony K.J.P., Jeyaraj M., Rathinam N.K., Gurunathan S. (2013). Biofabrication of gold nanoparticles and its biocompatibility in human breast adenocarcinoma cells (MCF-7). J. Ind. Eng. Chem..

[117] Alkilany A.M., Murphy C.J. (2010). Toxicity and cellular uptake of gold nanoparticles: What we have learned so far?. J. Nanopart. Res..

[118] Coradeghini R., Gioria S., García C.P., Nativo P., Franchini F., Gilliland D., Ponti J., Rossi F. (2013). Size-dependent toxicity and cell interaction mechanisms of gold nanoparticles on mouse fibroblasts. Toxicol. Lett..

[119] Patlolla A., Knighten B., Tchounwou P. (2010). Multi-walled carbon nanotubes induce cytotoxicity, genotoxicity and apoptosis in normal human dermal fibroblast cells. Ethn. Dis..

[120] Kumarathasan P., Breznan D., Das D., Salam M.A., Siddiqui Y., MacKinnon-Roy C., Guan J., de Silva N., Simard B., Vincent R. (2015). Cytotoxicity of carbon nanotube variants: A comparative: *In vitro* exposure study with A549 epithelial and J774 macrophage cells. Nanotoxicology.

[121] Chithrani B.D., Ghazani A.A., Chan W.C.W. (2006). Determining the size and shape dependence of gold nanoparticle uptake into mammalian cells. Nano Lett..

[122] Chithrani D.B. (2010). Intracellular uptake, transport, and processing of gold nanostructures. Mol. Membr. Biol..

[123] Oh N., Park J.H. (2014). Endocytosis and exocytosis of nanoparticles in mammalian cells. Int. J. Nanomed..

[124] Win K.Y., Feng S.-S. (2005). Effects of particle size and surface coating on cellular uptake of polymeric nanoparticles for oral delivery of anticancer drugs. Biomaterials.

[125] Nativo P., Prior I.A., Brust M. (2008). Uptake and intracellular fate of surface-modified gold nanoparticles. ACS Nano.

[126] Sahay G., Alakhova D.Y., Kabanov A.V. (2010). Endocytosis of nanomedicines. J. Control. Release.

[127] Moghimi S.M., Farhangrazi Z.S. (2014). Just so stories: The random acts of anti-cancer nanomedicine performance. Nanomedicine.

[128] Taurin S., Nehoff H., Greish K. (2012). Anticancer nanomedicine and tumor vascular permeability; Where is the missing link?. J. Control. Release.

[129] Toy R., Peiris P.M., Ghaghada K.B., Karathanasis E. (2014). Shaping cancer nanomedicine: The effect of particle shape on the *in vivo* journey of nanoparticles. Nanomedicine (Lond)..

[130] Kim K.Y. (2007). Nanotechnology platforms and physiological challenges for cancer therapeutics. Nanomedicine.

[131] Mirkin C., Meade Th.J., Petrosko S.H., Stegh A.H. (2015). Nanotechnology-Based Precision Tools for the Detection and Treatment of Cancer.

[132] Naguib Y.W., Cui Z. (2014). Nanomedicine: The promise and challenges in cancer chemotherapy. Adv. Exp. Med. Biol..

[133] Doane T.L., Burda C. (2012). The unique role of nanoparticles in nanomedicine: Imaging, drug delivery and therapy. Chem. Soc. Rev..

[134] Fubini B., Ghiazza M., Fenoglio I. (2010). Physico-chemical features of engineered nanoparticles relevant to their toxicity. Nanotoxicology.

[135] Kettiger H., Schipanski A., Wick P., Huwyler J. (2013). Engineered nanomaterial uptake and tissue distribution: from cell to organism. Int. J. Nanomed..

[136] Stone V., Johnston H., Schins R.P.F. (2009). Development of *in vitro* systems for nanotoxicology: methodological considerations. Crit. Rev. Toxicol..

[137] Hamburg M.A. (2012). Science and regulation. FDA’s approach to regulation of products of nanotechnology. Science.

[138] Desai N. (2012). Challenges in development of nanoparticle-based therapeutics. AAPS J..

